# Effectiveness of audiovisual interventions in increasing knowledge about sexually transmitted infections: a systematic review

**DOI:** 10.1590/1518-8345.7531.4704

**Published:** 2025-11-10

**Authors:** Emanoelle Fernandes Silva, Polyana Norberta Mendes, Marli Teresinha Gimeniz Galvão, Danielle Nedson Rodrigues de Macêdo, Breno Dias de Oliveira Martins, Rosilane de Lima Brito Magalhães

**Affiliations:** 1 Universidade Federal do Piauí, Teresina, PI, Brazil. Universidade Federal do Piauí PI Teresina Brazil; 2 Scholarship holder at the Conselho Nacional de Desenvolvimento Científico e Tecnológico (CNPq), Brazil. Scholarship holder at the Conselho Nacional de Desenvolvimento Científico e Tecnológico Brazil; 3 Centro Universitário Santo Agostinho, Teresina, PI, Brazil. Centro Universitário Santo Agostinho PI Teresina Brazil; 4 Universidade Federal do Ceará, Fortaleza, CE, Brazil. Universidade Federal do Ceará CE Fortaleza Brazil; 5 Universidade Federal do Piauí, Departamento de Enfermagem, Teresina, PI, Brazil. Universidade Federal do Piauí Departamento de Enfermagem PI Teresina Brazil; 6 Scholarship holder at the Universidade Federal do Piauí (UFPI), Brazil. Scholarship holder at the Universidade Federal do Piauí Brazil

**Keywords:** Adolescent, Adult, Aged, Audiovisual Aids, Video-Audio Media, Sexually Transmitted Diseases

## Abstract

to analyze the evidence on the effectiveness of audiovisual interventions in increasing knowledge about sexually transmitted infections in different age groups.

systematic review, conducted on February 21, 2023, in the Cumulative Index to Nursing and Allied Health Literature, Cochrane, Embase, Latin American and Caribbean Health Sciences Literature, Nursing Database, Spanish Bibliographic Index in Health Sciences, MEDLINE, Scopus, and Web of Science. Experimental studies that used audiovisual resources to improve knowledge were included, compared with other interventions or none. The synthesis of results was narrative, with assessment of risk of bias and certainty of evidence.

the sample consisted of 25 studies (10 randomized clinical trials and 15 quasi-experimental studies). Educational videos were the most widely used resource. Compared to other interventions or no intervention, the intervention group achieved significant improvements in knowledge in the medium and long term, as well as changes in perception and predisposition to reduce risky behavior. The certainty of the evidence was low for randomized studies and very low for quasi-experimental studies.

audiovisual interventions have shown effectiveness in different populations and age groups, highlighting the need to consider specific parameters in creating these resources.

## Highlights

**(1)** Effectiveness of audiovisual interventions in improving knowledge about STIs. **(2)** Changes in attitude and reduction in risky behaviors after using the intervention. **(3)** Usability of interventions for different populations. **(4)** Audiovisual resources are accepted as a tool for health promotion and prevention. **(5)** Use of theoretical models for the construction of interventions.

## Introduction

Sexually Transmitted Infections (STIs) have attracted considerable interest around the world in terms of combating and tackling them. International efforts have been made to achieve lower incidence rates for various STIs, such as syphilis, gonorrhea, hepatitis, and HIV/Aids^(1)^. Among these efforts, investment in innovative prevention and awareness actions stands out, such as providing health education to different populations.

There is evidence of low health literacy related to high-risk behavior for STIs in the general population^(2)^, which contributes to a lower perception of risk. On the other hand, when health literacy levels rise, there is an increase in the predisposition to adopt preventive behaviors, such as condom use^(3)^, and as a result, a reduction in STI cases.

In the context of health education, it is clear that Educational Technology (ET) is gaining ground as a resource for increasing health knowledge, with an emphasis on audiovisual resources. As the name suggests, these involve the combined use of images and sound. Examples include videos, slide presentations, animations, virtual reality, live broadcasts, holography, and interactive multimedia, among others^(4)^.

When applied in the context of health education, this type of intervention has had significant effects on improving health knowledge and perception^(5)^. These results are also consistent when applied specifically in the context of STIs^(6)^, demonstrating that there is evidence of the contribution of audiovisual resources in the provision of sexual health education.

Given this, it appears that consumption of videos on STI health issues has increased in recent years among adults, mainly through social media, while effectively promoting awareness and disseminating knowledge^(7)^. As audiovisual technology becomes increasingly present in everyday life, it is crucial to understand how these tools have been applied to health education, as well as the quality of interventions and their effects.

Thus, this review aims to analyze the evidence on the effectiveness of audiovisual interventions in increasing knowledge about STIs in different age groups.

It is hoped that valuable insights will emerge for healthcare professionals, educators, and intervention designers, contributing to the development of more effective strategies tailored to contemporary needs in sexual health prevention and promotion.

## Method

### Study design

This systematic review of effectiveness was conducted following the methodological steps developed by the JBI^(8)^ and reported in accordance with the Reporting Items for Systematic Reviews and Meta-Analyses (PRISMA)^(9)^. This study is registered in PROSPERO with ID CRD42022374619.

### Modifications to the protocol *a priori*

This review was conducted with the following modifications to the *a priori* protocol:

- The JBI standardized instruments for assessing risk of bias were not used; instead, the Cochrane Risk of Bias 2 (RoB 2) and Risk of Bias In Non-randomized Studies - of Interventions (ROBINS-I) tools were used. This decision was made based on the widespread recognition of their assessment in randomized and non-randomized studies. As the review included both, these tools were considered the most appropriate for an accurate and consistent assessment. In addition to being widely used in the literature, they are complementary to the principles of the JBI method and were applied rigorously and transparently without compromising the methodological integrity of the review;

- In addition to the primary outcome of knowledge, when present, the secondary outcomes of attitudes and behaviors were included;

- The data were extracted using an instrument created by the authors, based on the characteristics of the included studies;

- Only Randomized Controlled Trials (RCTs) and quasi-experimental studies were included. Previously, the inclusion of time series studies and analytical observational designs was anticipated. However, during the study selection and evaluation process, it was observed that RCTs and non-randomized trials would better serve the study objective, enabling a more direct and robust assessment of the effectiveness of interventions, as they make comparisons with pre-established groups, minimizing biases and confounding factors. Furthermore, the inclusion of studies with very heterogeneous designs could compromise the consistency of the results and the synthesis of the evidence.

### Review question

The question was formulated according to the PICO strategy, with P (Population: young people, adults, and the elderly); I (Intervention: audiovisual resources); C (Comparison: other interventions); O (Outcomes: knowledge about STIs). Thus, the following question was formulated: How effective are audiovisual interventions compared to other types of interventions in increasing knowledge about STIs among young people, adults, and the elderly?

### Eligibility criteria

RCTs and quasi-experimental studies were included. No direct searches were conducted in the gray literature. Thus, it was excluded due to variability in methodological quality and lack of formal peer review, which may compromise the reliability of the evidence.

The population included young people, adults, and the elderly^(10)^. It is important to note that not all included articles opted for specific age groups, but it was emphasized that there was no exclusion based on age. In addition, there were no restrictions regarding sex, education, income, race, or health status.

With regard to the type of intervention, only those that used audiovisual resources were considered, i.e., structured with the use of associated sound and images. This included educational videos, multimedia programs, documentary films, short films, and interactive DVDs. Interventions consisting only of audio calls without video, such as photo novels or radio soap operas, were excluded.

For the comparison of the effect of the main intervention, any other type of resource was considered, regardless of whether or not it was supported by audiovisual media.

The main outcome of this review was the level of knowledge about STIs, measured before and after the intervention (assessed by structured instruments). The secondary outcomes were those related to changes in behavior (physical actions or practices that contribute to prevention) and attitude (mental or emotional attitudes) toward STIs (measured by scales or self-reported accounts).

There were no restrictions on language or year of publication for the studies.

### Sources of information and search strategy

To verify the suitability of the review question, as well as the existence of completed or ongoing reviews on the topic, an initial search was conducted in PROSPERO, JBI Evidence Synthesis, and MEDLINE (PubMed). As no systematic reviews on the topic were found, a search was conducted using the main indexing terms in Medical Subject Headings (MeSH) and Health Sciences Descriptors (DeCS).

The databases searched were the Medical Literature Analysis and Retrieval System online (MEDLINE via PubMed), Scopus (via Elsevier), Web of Science (via Clarivate Analytics), Cumulative Index to Nursing and Allied Health Literature (CINAHL via EBSCOhost), Latin American and Caribbean Health Sciences Literature (LILACS via BVS), Spanish Bibliographic Index in Health Sciences (IBECS via BVS), Nursing Database (BDENF via BVS), Cochrane (via Wiley), and Embase (via Elsevier).

The strategy was initially developed in MEDLINE and subsequently adapted for each database according to its specifications and/or specific descriptors. The search strategy was then structured using a combination of appropriate terms, grouped by categories: population (young people, adults, and the elderly), interventions (audiovisual), and outcome (STIs). An initial search was conducted on January 11, and a final (update) search was conducted on February 21, 2023.

Thus, the search expression adopted is as follows: *# 1”Adolescent”[Mesh] OR Adolescent OR “Young Adult”[Mesh] OR (Young Adult) OR “Adult”[Mesh] OR Adult OR “Aged”[Mesh] OR Aged OR Adolescents OR Adolescence OR Teens OR Teen OR Teenagers OR Teenager OR Youth OR Youths OR (Adolescents, Female) OR (Adolescent, Female) OR (Female Adolescent) OR (Female Adolescents) OR (Adolescents, Male) OR (Adolescent, Male) OR (Male Adolescent) OR (Male Adolescents) OR (Adult, Young) OR (Adults, Young) OR (Young Adults) OR Adults OR Elderly AND #2 “Audiovisual Aids”[Mesh] OR (Audiovisual Aids) OR “Video-Audio Media” [Publication Type] OR (Video-Audio Media) OR “Webcast” [Publication Type] OR Webcast OR (Aid, Audiovisual) OR (Aids, Audiovisual) OR (Audiovisual Aid) OR (Audio-Visual Aids) OR (Aid, Audio-Visual) OR (Aids, Audio-Visual) OR (Audio Visual Aids) OR (Audio-Visual Aid) OR (Visual Aids) OR (Aid, Visual) OR (Aids, Visual) OR (Visual Aid) OR (Audiovisual Media) OR (Audio-Visual Media) OR Webcasts OR (Streaming Video) AND #3 “Sexually Transmitted Diseases”[Mesh] OR (Sexually Transmitted Diseases) OR “HIV”[Mesh] OR (HIV) OR “Syphilis”[Mesh] OR Syphilis OR “Hepatitis B”[Mesh] OR (Hepatitis B) OR “Chlamydia”[Mesh] OR Chlamydia OR “Gonorrhea”[Mesh] OR Gonorrhea OR “Trichomonas Infections”[Mesh] OR (Trichomonas Infections) OR “Herpes Genitalis”[Mesh] OR (Herpes Genitalis) OR “Papillomavirus Infections”[Mesh] OR (Papillomavirus Infections) OR (Disease, Sexually Transmitted) OR (Venereal Diseases) OR STDs OR (Sexually Transmitted Infections) OR (Infection, Sexually Transmitted) OR STIs OR STI OR (Human Immunodeficiency Virus) OR (Aids Virus) OR (Great Pox) OR (Hepatitis B Virus Infection).*

The searches and access to the databases were performed by two reviewers, using remote institutional access from the Federal University of Piauí (Teresina, Piauí, Brazil) through the Journal Portal of the Coordination for the Improvement of Higher Education Personnel (CAPES). The final list of references for the included articles was consulted, and the corresponding authors of articles that were not available in full were contacted when a means of communication was identified.

### Selection of studies

The retrieved studies were exported to EndNote (Clarivate Analytics, PA, USA) for duplicate removal. The data were then transferred to Rayyan software, where the selection stage took place through the reading of titles and abstracts, as well as the application of eligibility criteria.

Two independent reviewers, accompanied by a third party, participated in the screening process in a blinded manner. Eligible articles were set aside for full-text reading, followed by reapplication of the selection criteria. Those that did not meet the pre-established requirements were excluded, and the reasons were recorded. The results of the selection and inclusion process will be presented in full through the flowchart available in PRISMA^(9)^, in the results section.

### Data collection, variables, and effect measures

The data collection process was carried out in a structured manner, ensuring the integrity and accuracy of the information extracted from the studies included in the review. This step was also conducted by two independent reviewers who used a data collection form developed for this purpose. After initial extraction, the data were recorded in an organized spreadsheet to allow for comparisons and reviews. When there were doubts regarding the extraction of information between reviewers, a third reviewer was contacted for clarification.

The form used addressed the following variables: authors, year and place of publication, sample size, characteristics of participants (age, sex, race, education, and health status, if any), type of study, STI for which the intervention was intended, type of audiovisual resource used in the Intervention Groups (IG) and Control Group (CG), content of the intervention, type of instrument for pre- and post-test evaluation, follow-up time, outcome measures, effect measures for continuous and/or dichotomous data (odds ratio, mean difference, and Confidence Interval - CI), statistical significance, and other indicators of risk of bias (randomization, allocation concealment, blinding, completeness of the report).

It should be noted that the included studies provided the information necessary to complete the instrument qualitatively. In cases of quantitative data essential for knowledge assessment, in some studies, this information appeared in a generalized form, focusing only on statistical significance. The authors were contacted to request missing data, but no response was received. Some studies, due to the time of publication or the format of the journal itself, did not provide contact details for the corresponding author.

The effect was evaluated for each outcome, when available, using the mean difference after the final intervention, odds ratio, statistical significance, and CI.

### Risk of bias assessment

The risk of bias assessment was conducted for both randomized clinical trials and quasi-experimental studies using the tools provided by Cochrane: the Revised Cochrane Risk-of-Bias tool for Randomized Trials (RoB 2)^(11)^ and the Risk Of Bias In Non-randomized Studies - of Interventions (ROBINS-I)^(12)^, respectively. ROB 2 assesses five domains, which are allocation bias, performance bias, detection bias, reporting bias, and selection bias. ROBINS-I covers seven domains, including confounding bias, participant selection bias, intervention classification bias, protocol deviation bias, data collection bias, missing data bias, and selective reporting bias.

This process was performed by two independent reviewers and is presented in detail in the results section. Regardless of methodological quality, all studies underwent the process of synthesis and data extraction and were subsequently included in this review.

### Summary of results

Due to the variability and heterogeneity of the included articles, as well as the lack of complete quantitative data, it was not possible to perform a meta-analysis. Most studies reported results qualitatively, without providing sufficient numerical values to allow statistical combination of the data.

In addition, the lack of standardization in the outcomes, measured by different knowledge instruments, and the incomplete description of audiovisual interventions in some studies limited the comparison between studies. Thus, the data synthesis is presented in a narrative form.

To facilitate the comparative analysis of the results, the studies were grouped according to the type of study. Each was organized by type of audiovisual intervention (educational videos, multimedia programs, DVDs), knowledge assessment instrument, and measured outcomes, including increased knowledge about STIs and changes in attitudes and behaviors.

The Grading of Recommendations Assessment, Development and Evaluation Working Group (GRADE)^(13)^ was adopted, using GRADEpro software^(14)^, to assess the certainty of the evidence. The analysis was performed for each outcome analyzed, according to the type of study.

## Results

### Inclusion of studies

**Figure 1-:**
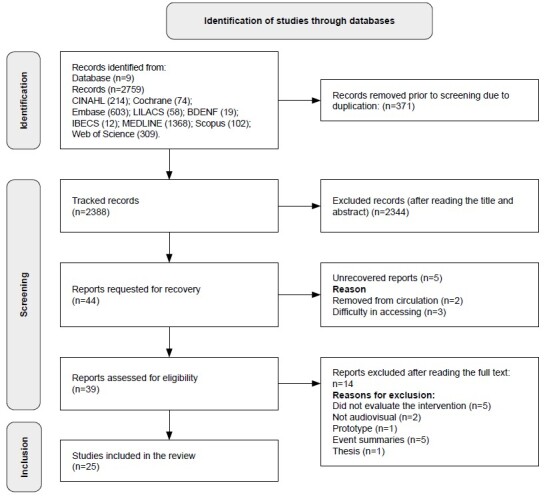
Flowchart of the selection process for studies included in the systematic review, adapted from the Preferred Reporting Items for Systematic Reviews and Meta-Analyses (PRISMA) (n = 25). Teresina, PI, Brazil, 2024

Source: Adapted^(9)^

In this study, the search for evidence was conducted by consulting electronic databases and, additionally, by manually searching the reference lists of the included studies. However, the manual search did not result in the identification of new studies eligible for inclusion in the review. Thus, we chose to adapt the PRISMA flowchart, excluding the step “Identification of studies by other methods,” since it did not add anything to the selection process (Figure 1).

The database searches resulted in 2,759 studies; of these, 371 were removed due to duplication. Next, 2,388 articles were selected for reading titles and abstracts. Applying the selection criteria, the final sample consisted of 25 studies, including 10 RCTs and 15 quasi-experimental studies. The selection process and reasons for exclusion are detailed in Figure 1.

During the full-text reading, five studies could not be retrieved. Of these, two were withdrawn from circulation, and three remained inaccessible in full, even after attempts to contact the corresponding authors.

In addition, the LILACS search retrieved a doctoral thesis that addressed the same educational intervention presented in one of the articles included in this review. However, after careful evaluation, it was found that the thesis had not been published in a peer-reviewed journal. Therefore, it was decided to exclude it to maintain methodological consistency and predefined eligibility criteria. Additional details about the thesis and its relationship to the published study are described in the discussion section.

### Risk of bias assessment

**Figure 2-:**
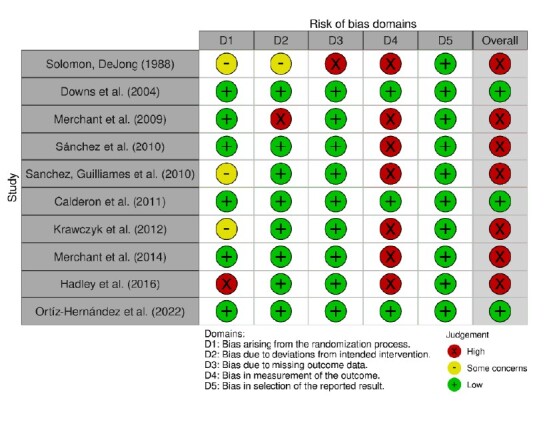
Assessment of risk of bias in randomized controlled trials in each domain of the RoB 2 tool (n = 10). Teresina, PI, Brazil, 2024

Source: Image created by Robvis software^(15)^

The assessment of bias risk in the studies varied, indicating different levels of certainty about the validity of the results obtained. In the case of RCTs, three studies were found to have a low risk of bias, while seven had a high risk. Among these, one study showed a high risk of bias due to the randomization process (D1), as randomness was not guaranteed; one due to deviations from the intended interventions (D2), as participants were aware of the intervention received; and one due to a lack of outcome data (D3), evidenced by significant loss of participants during follow-up. In addition, seven studies revealed a high risk of bias in outcome measurement (D4), as the outcome assessors were aware of the intervention applied to the IG and CG (Figure 2).

The assessment of bias risk in the studies varied, indicating different levels of certainty about the validity of the results obtained. In the case of RCTs, three studies were found to have a low risk of bias, while seven had a high risk. Among these, one study showed a high risk of bias due to the randomization process (D1), as randomness was not guaranteed; one due to deviations from the intended interventions (D2), as participants were aware of the intervention received; and one due to a lack of outcome data (D3), evidenced by significant loss of participants during follow-up. In addition, seven studies revealed a high risk of bias in outcome measurement (D4), as the outcome assessors were aware of the intervention applied to the IG and CG (Figure 2).

**Figure 3-:**
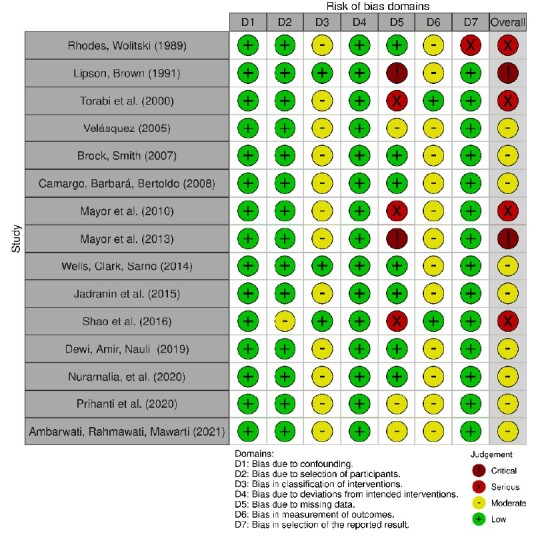
Assessment of bias risk in quasi-experimental studies in each domain of the ROBINS-I tool (n = 15). Teresina, PI, Brazil, 2024

Source: Image created by Robvis software^(15)^

### Setting and year of included studies

The studies included varied in year of publication, with the oldest study dating from 1988^(16)^ and the most recent from 2022^(6)^. Regarding the locations where the research was conducted, 13 (52%) were conducted in the United States^(16-28)^, four (16%) in Indonesia^(29-32)^, and one (4%) in each of the following countries: Russia^(33)^, Peru^(34)^, Brazil^(35)^, Canada^(36)^, Puerto Rico^(37)^, Serbia^(38)^, Mexico^(6)^, and unspecified (online study)^(39)^.

### Participants

The 25 studies analyzed totaled 6,021 participants, of which 2,176 belonged to the 10 RCTs^(6,16,19,21,23-25,27-28,37)^ and 3,845 to the quasi-experimental studies^(17-18,20,22,26,29-35,37-39)^.

Significant variations were observed among the participants. Three studies were conducted with university students^(17-18,37)^, while eight studies focused on adolescents, with ages ranging from ≤ 13 years to 21 years^(19,25,28,30,32-36)^, one of which also addressed caregivers^(28)^. Four studies were conducted with people living with an STI^(16,20,22,37)^, and one study was conducted with men with mild to moderate intellectual disabilities^(26)^.

Adult participants treated in an emergency department^(21)^, men who have sex with men^(23)^, Spanish-speaking Latinos^(27)^, Internet users^(39)^, armed forces soldiers^(38)^, health professionals^(31)^, and young people over 18 years of age^(6)^ were also included. It is worth noting that most studies focused exclusively on males^(16,23-24,26,38)^, while two were conducted with females^(19,29)^. No studies conducted exclusively with elderly people were identified; the oldest age reported in the general public was 70 years^(20)^.

### Interventions/comparators

The interventions applied will be addressed according to the type of study. Among RCTs, the most widely adopted modality in the IG was the use of videos. Five of them relied solely on the use of educational videos, focusing on providing information about gonorrhea (It Just Happens Sometimes)^(16)^, HIV/Aids (Do you know about rapid HIV testing?)^(21)^; educational video in Spanish about HIV/Aids^(27)^, syphilis (Syphilis and Men)^(23-24)^, and HPV (*7 cosas que debes saber sobre el VPH*!)^(6)^. One video was interactive about STIs in general^(19)^, providing not only information but also elements that allowed participants to take action.

In addition, one video focused on education and pre-test counseling for HIV^(25)^. Another intervention combined the use of an educational pamphlet and video to address HPV and vaccination^(36)^. Finally, there was an intervention that used a DVD in conjunction with an activity book^(28)^, providing a more comprehensive approach. This covered several modules to promote communication, prevention, and interpersonal skills related to HIV/Aids (Work It Out Together).

The duration of the interventions also varied, with most lasting less than 10 minutes. The shortest duration recorded was three minutes^(6)^, while the longest was one hour^(19)^.

Among the comparators, CG, it is noteworthy that two received no intervention^(6,16)^, one received standard care without undergoing an intervention^(23)^, two received reading materials^(19,36)^, three underwent face-to-face counseling^(21,25,27)^, one received a palm card (part of a social marketing campaign in response to the syphilis epidemic)^(24)^, and one was shown a DVD promoting general health that did not focus specifically on reducing risks related to sexual behavior or substance use^(28)^. The summary of RCTs by type of intervention is shown in Figure 4.

In quasi-experimental studies, video was also the most widely used modality, including informational, educational, and documentary videos. Three interventions were based on the use of multimedia educational programs^(22,37)^, one of which was interactive and accessible to people with intellectual disabilities^(26)^. One intervention adopted the format of a short film^(38)^, while another involved a government program that used audiovisual media applied through discussion sessions on the theme of HIV/Aids^(30)^.

There was a wide variation in the duration of the interventions, with some studies not providing a detailed description. The shortest duration recorded was 15 minutes^(39)^, while the longest reported duration was two hours^(33)^. Regarding the CG, nine studies did not include a CG, four did not apply any intervention in the comparison group, and only three compared the main intervention with another (Figure 5).

Few studies described the means of transmission through which the interventions were made available to participants, how the intervention was actually structured, whether they were accessible physically or electronically, and whether they contained elements of accessibility for populations with disabilities. More information about the interventions is available in Figures 4 and 5

The theoretical frameworks used in the intervention reported in the studies included: Bandura’s social learning theory^(26)^, Health Belief Model (HBM)^(16,22,37)^, Social Cognitive Theory (SCT)^(22,37)^, Health media and marketing^(24)^, theoretical model of symbolic mental models and cognitive rehearsal^(19)^, Albion and Gibson’s problem-based and critical thinking constructivist methodology^(34)^, Health Promotion Theory, Theory of Planned Behavior, and Health Belief Theory^(36)^, and Fisher and Fisher’s Model of Information, Motivation, and Behavioral Skills^(6)^.

### Outcome

The studies covered a range of STIs, with 14 studies focusing on HIV/Aids^(17-18,20-21,25-27,29-32,33,35,38-39)^, while two studies addressed HIV and STIs in combination^(28,34)^. Two studies dealt with hepatitis C virus^(22,37)^, one study addressed STIs in general^(19)^, one study addressed gonorrhea^(16)^, two studies dealt with syphilis^(23-24)^, and two studies dealt with HPV^(6,36)^. HIV has been the most explored STI in the context of audiovisual educational interventions (Figures 4 and 5).

Regarding the primary outcome, all studies assessed knowledge using instruments applied before and after the intervention, and there was a significant improvement (p<0.05) in this variable in the post-test for the treatment groups; however, two randomized controlled studies did not show significant changes after the intervention^(6,27)^. And among the quasi-experimental studies, despite statistically significant changes in knowledge scores, two studies assessed that the magnitude of these changes was modest^(17-18)^ (Figures 4 and 5).

There was no description in the studies of how the format in which the interventions were created impacted knowledge improvement. Only one study reported that a more scientific presentation approach showed better results in knowledge assessment than a more informal one^(35)^.

Only 11 studies addressed outcomes other than knowledge. Most involved intervention in attitude changes, with notable improvements in adherence to HIV testing^(25)^, HPV vaccination^(36)^, reduction in risky sexual behaviors^(18-19,28)^, and substance use^(28)^. There was an improvement in perceived self-efficacy in relation to treatment adherence^(20)^, improvement in HIV/Aids-related skills and condom use^(26)^, increased HIV protection behavior^(29)^, and changes in attitude toward HIV/Aids^(31-32)^. No changes were found in the predisposition to notify sexual contacts about STI diagnosis^(16)^. Only one study addressed behavioral changes, focusing on reducing the sharing of syringes and drug preparation equipment^(37)^ (Figures 4 and 5).

**Figure 4-:** Summary of included randomized controlled clinical trials (n = 10). Teresina, PI, Brazil, 2024

**Authors, year, and location**	**Sample/follow-up time**	IG*/type of educational technology	**CG** ^†^ **/type of technology**	**Knowledge assessment tool**	**Key findings**
Solomon, DeJong. 1988. USA ^( 16 )^	n=175/14 days	**Video:** “It Just Happens Sometimes” (10 min; n=83) Type/STI ^‡^ : Educational video/Gonorrhea Content: Characters discussing symptoms, treatment, and prevention, with an emphasis on counseling for diagnosis. Target audience: Black men with gonorrhea.	Group without educational video (n=92).	18-item true/false test.	**Knowledge:** Average number of correct answers IG* 16.6 (sd ^§^ = 1.7), CG ^†^ 13.3 (sd ^§^ = 2.2; 1(239) =13.4, p ^||^ <0.0001). **Attitude** : Willingness to notify sexual contacts (p ^||^ >0.05) between IG* and CG ^†^ .
Downs, et al. 2004. USA ^( 19 )^	n=300/6 months	**Video:** (1 h; n= not explicit) Type/STI ^‡^ : Interactive video / STI ^‡^ Content: Negotiation behaviors, effective condom use, female reproductive anatomy, and physiological responses to infections. Target audience: Female adolescents.	Reading materials (n=not explicit).	40-item true/false test.	**Knowledge:** Mean difference between IG* and CG ^†^ , 5.3 points and 5.1 points, respectively. Significant improvements in knowledge over time (overall: F ^¶^ (1,214= 69.09, p ^||^ <0.001; specific: F ^¶^ (1,214)= 58.62, p ^||^ <0.001).
Merchant, et al. 2009. USA ^( 21 )^	n=574/no follow-up	**Video:** “Do you know about rapid HIV ** testing?” (9.5 min; n=270) Type/STI ^‡^ : Educational video/HIV** Content: Definition, transmission, prevention, and testing. Target audience: Adults who visited an urgent care center or emergency room.	Face-to-face session with HIV** testing counselor (n=304).	26-question “Rapid HIV** Pretest Information Comprehension” questionnaire.	**Knowledge:** Mean difference IG* vs. CG ^†^ = 0.68 (CI ^††^ 95%; 0.18-1.26; p ^||^ <0.05). The mean scores differed by less than one correctly answered question.
Sánchez, et al. 2010. USA ^( 23 )^	n=206/ no follow-up	**Vídeo:** “Syphilis and Men” (5 min; n=100) Type/STI ^‡^ : Educational video/Syphilis Content: Transmission, symptoms, treatment, connection between syphilis and HIV**, and risk reduction. Target audience: Men who visited an urgent care center or emergency room.	Standard care without video on syphilis (n=106).	Questionnaire with 10 multiple-choice questions.	**Knowledge:** IG* scored on average 24.8% higher than CG ^†^ in the post-intervention test (p ^||^ < 0.001). IG* scored on average 24.8% higher than CG ^†^ in the post-intervention test (p ^||^ < 0.001).
Sánchez, Guilliames, et al. 2010. USA ^( 24 )^	n=168/no follow-up	**Video:** “Syphilis and Men” (5 min; n=85) Type/STI ^‡^ : Educational video/Syphilis Content: Transmission, symptoms, treatment, connection between syphilis and HIV**, and risk reduction. Target audience: Black men who have sex with men.	“Syphilis is Back” Palm Card (n=83).	A questionnaire developed by researchers (10 multiple-choice questions).	**Knowledge:** IG* 8.9 to 9.8 correct answers, CG ^†^ 6.9 to 7.9 correct answers. Increase from 19.5% to 20.9% in the post-test score for IG* (p ^||^ <0.05).
Calderon, et al. 2011. USA ^( 25 )^	n=200/no follow-up	**Video:** (4 min, n=100) Type/STI ^‡^ : Educational video/HIV** Content: Covers seven essential elements for a pre-test counseling session for HIV**. Target audience: Adolescents.	Face-to-face session with HIV testing counselor** (n=100).	10-question survey created by Carey and Schroder, and questions from the New York State Department of Health.	**Knowledge:** Mean difference IG* vs. CG ^†^ = 12.2% (p ^||^ <0.01); IG* 78.5% correct and CG ^†^ 66.3% correct. **Attitude** : Accepting HIV testing**: IG*, OR ^‡‡^ = 3.6x (CI ^††^ 95%:1.8–7.2; p ^||^ <0.001) compared to CG ^†^ .
Krawczyk, et al. 2012. Canada ^( 36 )^	n=200/no follow-up	**Video** (5 min, n=74) **+ Educational pamphlet** (n=61) Type/STI ^‡^ : Video + Pamphlet/HPV ^§§^ Content: The video features a doctor discussing the incidence, transmission, consequences of the virus, and effectiveness of the vaccine. The pamphlet provides information about HPV ^§§^ and the vaccine. Target audience: College students.	Only pamphlet on cancer prevention in general (n=65).	22-item test (evaluation: true, false, and don’t know).	**Knowledge:** Average correct answers IG* (pamphlet) = 17.46; IG* (video) = 16.70; CG ^†^ = 12.06 (p ^||^ <0.001). **Attitude** : Increase in vaccination intention for IG* (pamphlet) and IG* (video) compared to CG ^†^ (p ^||^ < 0.001).
Merchant, et al. 2014. USA ^( 27 )^	n=150/ no follow-up	**Video:** (9.5 min, n=75) Type/STI ^‡^ : Educational video/HIV** Content: Definitions, nature, transmission, prevention, testing methods, and interpretation of HIV** results. Target audience: Spanish-speaking Latinos.	Oral information from HIV testing counselor** (n=75).	25-item test (Assessment: yes, no, don’t know).	**Knowledge:** Mean difference IG* vs. CG ^†^ = IG* 20.4 (95% CI ^††^ : 19.5-21.3); CG ^†^ 20.6 (95% CI ^††^ : 19.7-21.5), indicating similarity (95% CI ^††^ :-1.4-1.1).
Hadley, et al. 2016. USA ^( 28 )^	n=170/3 months	**DVD + book** : “Work It Out Together” (20 min/module; n=83) Type/STI ^‡^ : Interactive DVD + activity book/HIV** Content: Six modules addressing communication between parents and adolescents, prevention, condom use, negotiating safe sex, skills, and decision-making. Target audience: Adolescents.	General health promotion DVD (n=87).	Knowledge test with 10 items (true, false, uncertain).	**Knowledge:** Average correct answers IG*= 6.71; CG ^†^ = 6.45. Parents scored higher (t ^||||^ (158)=2.00, p ^||^ <0.05). **Attitude** : IG* (parents) greater self-efficacy to engage in preventive behaviors (t ^||||^ (158)= 2.28, p ^||^ <0.05). **Attitude** : IG* (adolescents) greater self-efficacy to use condoms (t ^||||^ (64)= 2.06, p ^||^ <0.05).
Ortíz, et al. 2022. Mexico ^( 6 )^	n=33/7 days	**Vídeo:** “7 cosas que debes saber sobre el VPH ^§§^ !” (3 min; n=16) Type/STI ^‡^ : Educational video/HPV ^§§^ Content: Definition, forms of transmission, symptoms, diagnosis, treatment, and prevention. Target audience: Young people over the age of 18.	Group without educational video (n=17).	38-item test (True, False, and Don’t Know).	**Knowledge:** IG* higher than CG ^†^ (p ^||^ =0.020 and p ^||^ =0.024, respectively). Average correct answers IG*= 30.75 (sd ^§^ = 2.32); CG ^†^ 28.18 (sd ^§^ = 3.55).

*IG = Intervention Group; ^†^CG = Control Group; ^‡^STI = Sexually Transmitted Infections; ^§^SD = Standard Deviation; ^||^p = Statistical Significance; ^¶^F = Analysis of Variance; **HIV = Human Immunodeficiency Virus; ^††^CI = Confidence Interval; ^‡‡^OR = Odds Ratio; ^§§^HPV = Human Papillomavirus; ^||||^t = t-statistic (Student’s t-test)

**Figure 5-:** Summary of quasi-experimental studies included (n = 15). Teresina, PI, Brazil, 2024

**Authors, year, and location**	**Sample/follow-up time**	IG*/type of educational technology	**CG** ^†^ **/type of technology**	**Knowledge assessment tool**	**Key findings**
Rhodes, Wolitski. 1989. USA ^( 17 )^	n=441/6 weeks	**Videos:** (16-20 min; n= not explicit) Type/STIs ^‡^ : 4 Informational videos/Aids Content: (1) Aids: Acquired Immune Deficiency Syndrome, 1986, 18 min; (2) Aids: What Everyone Needs to Know, 1986, 18 min; (3) Beyond Fear: The Virus, 1986, 20 min; (4) Sex, Drugs, and Aids, 1986, 16 min. Target audience: University students.	Did not watch any of the videos (n= not explicit).	True and false test with 23 items.	**Knowledge:** Increase in knowledge in the four videos (p ^§^ <0.001), superior to CG ^†^ in the late post-test (p ^§^ <0.01).
Lipson, Brown. 1991. USA ^( 18 )^	n=144/1 month	**Video:** (19-40 min; n=90) Type/STIs ^‡^ : 3 Informative Videos/Aids Contents: (1) Sex, Drugs, and Aids, 19 min; (2) Aids: Beyond Fear, 40 min; (3) Aids: What You Need to Know, 40 min. Target audience: University students.	The video “Where Did I Come From?” addresses sex education, but not HIV/Aids (n=54).	55-item questionnaire (The most correct answer received a score of 4, and the least correct answer received a score of 0).	**Knowledge:** Average difference IG* vs. CG ^†^ = 15.93 points (p ^§^ <0.01). **Attitude** : IG* improvements in attitudes toward preventive behaviors, average difference of 1.33 points compared to CG ^†^ (p ^§^ <0.01).
Torabi, et al. 2000. Russia ^( 33 )^	n=1.124/2 weeks	**Video:** (2 h; n=731) Type/STI ^‡^ : Informational video/HIV ^||^ Content: Prevention, using lecture techniques, illustrations, and questions and answers. Target audience: Adolescents.	Usual academic program, excluding education about HIV/Aids (n=393).	51-question test (“yes,” “no,” and “not sure”).	**Knowledge:** IG* scores were higher than CG ^†^ scores (F ^¶^ =58.73, p§<0.01).
Velásquez. 2005. Peru ^( 34 )^	n=454/there was no follow-up	**CD:** “ *Planeta Riesgo Xero* ” (duration not specified; n=454) Type/STI ^‡^ : Educational CD/STI ^‡^ and HIV ^||^ Content: Everyday stories of adolescents, game about decision-making, general information, risks, myths, and beliefs. Target audience: Adolescents.	There was no CG.	Questionnaire with 101 questions divided into four sections.	**Knowledge:** 3x increase in knowledge about HIV ^||^ and 6x increase in knowledge about Aids ( *p* ^§^ <0.001). Increased likelihood of knowing about gonorrhea (OR**=5.4), syphilis (OR**=4.8), chlamydia (OR**=13.2), genital herpes (OR**=3.7), and soft chancre (OR**=2.3, *p* ^§^ <0.001).
Brock, Smith. 2007. USA ^( 20 )^	n=51/4 to 6 weeks	**Video:** (25 min; n=51) Type/STI ^‡^ : Educational video/HIV ^||^ Content: Shown on a Personal Digital Assistant (PDA), containing information on adherence to antiretroviral medication. Target audience: Infectious disease outpatient clinic patients.	There was no CG.	Instrument with nine questions.	**Knowledge** : Improvements in knowledge of HIV ^||^ and treatment ( *p* ^§^ <0.005). Attitude: Increase in self-efficacy for treatment adherence ( *p* ^§^ <0.005).
Camargo, Barbará, Bertoldo. 2008. Brazil ^( 35 )^	n=141/1 week	**Videos:** (duration not specified) Type/STI ^‡^ : Documentary videos/HIV ^||^ Content: Video 1, scientific approach, development of Aids, prevention, and details of the disease (n=46). Video 2, basic information and testimonials from adolescents living with the virus (n=39). Target audience: Adolescents.	Did not watch any of the videos (n=56).	Scientific knowledge test on Aids.	**Knowledge:** IG*1 showed better knowledge than IG*2 and CG ^†^ (F ^¶^ =8.15; p ^§^ <0.001).
Mayor. 2010. USA ^( 22 )^	n=110/8 weeks	**Multimedia program:** (25-35 min/session; n=110) Type/STI ^‡^ : Educational program/HCV ^††^ Content: Developed in PowerPoint, it addresses susceptibility, protective measures, and the relationship with HIV ^||^ .	There was no CG.	Self-administered questionnaire with Likert scale.	**Knowledge:** Understanding that hepatitis C is a viral condition increased from 73.1% to 85.2% (p ^§^ =0.03). Understanding that HCV ^††^ is predominantly found in blood increased from 92% to 98.2% (p ^§^ =0.01).
Mayor. 2013. Puerto Rico ^( 37 )^	n=88/6 months	**Target audience:** Patients living with HIV ^||^ . Multimedia program: (duration not specified; n=88) Type/STI ^‡^ : Educational program/HCV ^††^ Content: Developed in PowerPoint, it addresses susceptibility, protective measures, and the relationship with HIV ^||^ . Target audience: Drug users with HIV ^||^ .	There was no CG.	Self-administered questionnaire with Likert scale.	**Knowledge:** Increased awareness of clinical manifestations, risk behaviors, prevention practices, and synergisms of HIV ^||^ /HCV ^††^ coinfection (p ^§^ <0.05). **Behavior** : Reduction in sharing of syringes and drug preparation equipment from 39.8% to 13.6% (p ^§^ <0.001).
Wells, Clark, Sarno. 2014. USA ^( 26 )^	n=49/ there was no follow-up	**Multimedia program:** (1.5 hours, n=49) Type/STI ^‡^ : Interactive program/HIV ^||^ Content: Computer-based, focused on prevention. Target audience: Men with mild to moderate intellectual disabilities.	There was no CG.	Questionnaire with 44 questions.	**Knowledge:** Gains in all areas evaluated, with an average effect size of 0.67 (large effect).
Jadranin. 2015. Serbia ^( 38 )^	n=102/ no follow-up	**Film:** “HIV/Aids prevention and control in the Serbian Armed Forces” (17 min, n=102) Type/STI ^‡^ : Educational film/HIV ^||^ Content: Prevention, transmission, disease progression, and testing. Target audience: Armed forces soldiers.	There was no CG.	23-question multiple-choice questionnaire.	**Knowledge:** Average difference before and after = 1.91 points, with an increase in score after the intervention (p ^§^ <0.001).
Shao. 2016. Online study ^( 39 )^	n=817/6 weeks	**Video:** (15 min, n=817) Type/STI ^‡^ : Informative video/HIV ^||^ Content: Uses animation to provide information about acute infection and testing methods. Target audience: Internet users.	There was no CG.	Questionnaire with 25 questions + a single question on a 4-point scale.	**Knowledge:** Average difference before and after the video = English speakers (Post: 19.6 vs. Pre: 16.4; Δ ^‡‡^ =3.2; CI ^§§^ 95%: 2.8-3.5) and Spanish speakers (Post: 20.7 vs. Pre: 17.3; Δ ^‡‡^ =3.4; CI ^§§^ 95%: 3.0-3 .8), with English speakers showing less prior knowledge (p ^§^ <0.05).
Dewi, Amir, Nauli. 2019. Indonesia ^( 29 )^	n=144/ no follow-up	**Video + brochures:** (n=72) Type/STI ^‡^ : Educational video + brochure/HIV ^||^ Content: Information to increase understanding and promote preventive behaviors. Target audience: Married women.	Did not receive any of the interventions (n=72).	Questionnaire tested for validity and reliability.	**Knowledge:** Mean difference between IG* vs. CG ^†^ = 3.1; IG*(12.86) and CG ^†^ (9.69) (95% CI: 2.60-3.73, p ^§^ = 0.000). Attitude: Mean difference of 8.4 for preventive attitude between IG* and CG ^†^ (95% CI: 0.879-6.66, p ^§^ =0.000).
Nuramalia, et al. 2020. Indonesia ^( 30 )^	n=96/4 weeks	**ABAT audiovisual media:** “I’m Proud I Know” (120 min/session; n=48) Type/ STI ^‡^ : Educational media/HIV ^||^ Content: Reproductive health, drugs, lifestyle, HIV ^||^ and Aids. Target audience: Adolescents.	One session per week (lasting three weeks), covering the same topics (n=48).	Questionnaire with questions on knowledge and prevention practices.	**Knowledge:** Increase in the “Good” category to 91.6%, while the “Sufficient” category was 8.3%. There were differences in knowledge between IG* and CG (p ^§^ < 0.05).
Prihanti. 2020. Indonesia ^( 31 )^	n=41/ no follow-up	**Video:** (duration not specified; n=41) Type/STI ^‡^ : Online video/HIV ^||^ Content: Contains information about the general picture of HIV, risk factors, transmission, and the importance of diagnostic testing. Target audience: Health professionals.	There was no CG.	E-Questionnaire via Google Form with 28 questions.	**Knowledge:** 97.6% of participants demonstrated good knowledge and only 2.4% demonstrated insufficient knowledge (p ^§^ =0.00). **Attitude** : 85.4% of participants demonstrated positive attitudes and 14.6% demonstrated negative attitudes (p ^§^ =0.00).
Ambarwati. 2021. Indonesia ^( 32 )^	n=43/ no follow-up	**Vídeo + slides:** (n=43) Type/STI ^‡^ : Educational video + slides/HIV ^||^ Content: Developed to improve knowledge and attitudes towards HIV ^||^ /Aids. Target audience: Adolescents.	There was no CG.	Questionnaire with non-detailed questions.	**Knowledge:** Increase in knowledge (Z ^||||^ =-3.819; p ^§^ =0.000). **Attitude** : Increase in attitude after the intervention (Z ^||||^ =-3.873; p ^§^ =0.00).

*IG = Intervention Group; ^†^CG = Control Group; ^‡^STI = Sexually Transmitted Infections; ^§^p = Statistical Significance; ^||^HIV = Human Immunodeficiency Virus; ^¶^F = Analysis of Variance; **OR = Odds Ratio; ^††^HCV = Hepatitis C Virus; ^‡‡^Δ = Delta Statistic; ^§§^CI = Confidence Interval; ^||||^Z = Z Statistics

The assessment of the certainty of the evidence according to the selected outcomes is presented in Figure 6. The quality of the evidence was classified as low for RCTs and very low for quasi-experimental studies, due to factors such as serious or extremely serious risk of bias, imprecision (wide confidence intervals or lack of quantitative data), and non-serious inconsistency. Most studies had methodological limitations, such as a high risk of bias and imprecision, which impacted the reliability of the results.

**Figure 6-:** Assessment of the certainty of evidence, according to the Grading of Recommendations Assessment, Development and Evaluation (GRADE). Teresina, PI, Brazil, 2024

**Certainty of evidence**							**Number of patients**		**Effect** ^††^		**Certainty**
**Number of studies**	**Study design**	**Risk of bias**	**Inconsistency**	**Indirect evidence**	**Inaccuracy**	**Other considerations**	**Audiovisual resources**	**Neither one nor the other**	**Relative** (95% CI*)	**Absolute** (95% CI*)	
**Increased knowledge about STIs**											
10	RCT ^†^	serious ^‡^	not serious	not serious	serious ^§^	none	886/1815 (48.8%)	929/1815 (51.2%)	not estimable	not estimable	⨁⨁◯◯ Low ^‡§^
15	Quasi-experimental studies	extremely serious ^||^	not serious	not serious	serious ^§^	none	2781/3404 (81.7%)	623/3404 (18.3%)	not estimable	not estimable	⨁◯◯◯ Very low ^§||^
**Change of attitude**											
4	RCT ^†^	serious ^¶^	not serious	not serious	serious ^§^	none	327/671 (48.7%)	344/671 (51.3%)	not estimable	not estimable	⨁⨁◯◯ Low ^§¶^
5	Quasi-experimental studies	extremely serious**	not serious	not serious	serious ^§^	none	297/423 (70.2%)	126/423 (29.8%)	not estimable	not estimable	⨁◯◯◯ Very low ^§^ **
**Behavior change**											
1	Quasi-experimental studies	extremely serious**	not serious	not serious	serious ^§^	none	88/88 (100.0%)	0/0	not estimable	not estimable	⨁◯◯◯ Very low ^§^ **

*CI = Confidence Interval; ^†^RCT = Randomized Controlled Trial; ^‡^Seven studies presented a high risk of bias according to the ROB-2 tool; ^§^Some results presented wide CIs, indicating imprecision. For other results, there is a lack of quantitative data, which prevents a complete assessment of precision; ^||^Two studies presented a critical risk of bias according to the ROBINS-I tool; ^¶^Three studies present a high risk of bias according to the ROB-2 tool; **One study presents a critical risk of bias according to the ROBINS-I tool; ^††^Not estimable due to the variability of the studies, which made meta-analysis impossible

## Discussion

The objective of this systematic review was to analyze the available evidence on the effectiveness of audiovisual interventions in increasing knowledge about STIs among young people, adults, and the elderly. Based on the results, it was possible to show that these resources contribute to a homogeneity of factual knowledge, especially among adolescents and young adults. These populations are considered at risk for STIs, since more than half (56%) of new HIV cases are concentrated in the 13-34 age group^(40)^. Therefore, interventions have focused on the segment of the population that most needs access to information on the subject.

Most of the studies included pointed out that audiovisual educational interventions have been widely explored as effective tools in promoting knowledge about STIs, especially HIV/Aids. In addition, these technologies have an impact on changing attitudes and, in some cases, reducing risk behaviors related to these infections.

It should be noted that audiovisual technologies, as they are considered part of Information and Communication Technologies (ICT), are also classified as “light-heavy technologies”. In other words, they are presented through hardware and devices and require other resources, such as cameras, microphones, speakers, monitors, and software related to the production and reproduction of audiovisual content^(41)^.

This type of “light-heavy” technology has the potential to stimulate learning and behavioral change in health, as it enables an approach that stimulates the senses and keeps stakeholders more firmly focused on the content they intend to present^(42)^. It can be inferred that interventions supported by this type of classification can have satisfactory effects in increasing health knowledge and the adoption of safer practices and behaviors.

As described, there was consensus among the publications regarding the impact of audiovisual media on knowledge. In general, it is possible to see that the information conveyed was well absorbed by the participants in the post-test^(19)^. In addition, the influence of educational videos on significantly improving knowledge about HIV/Aids in North American communities where Spanish is dominant is also noteworthy^(27)^.

However, despite the large number of outcomes showing statistically significant improvements in knowledge, it is also necessary to consider that the strength of these changes may be modest^(17)^. In view of this, clinical relevance should be considered in addition to statistical significance. In other words, there is a need to observe this improvement in knowledge together with the participants’ predisposition to reduce risky behaviors and adopt preventive measures in relation to STIs.

The diversity of contexts in which the studies were conducted, including different populations (directly and indirectly involved in the context of STIs), age groups, and levels of health literacy, highlights the flexibility and adaptability of educational video interventions in health promotion. However, the generalization of the results to the general population may be limited, especially when the target population is in a school setting and has literacy levels considered satisfactory for their age^(35)^.

Few studies have looked at whether the participants’ level of education can interfere with the learning process. A dynamic video approach, such as one that tells a character-based story about gonorrhea in the context of negotiating with partners about STI reporting and testing, led all patients in the treatment group to a high level of knowledge, regardless of educational level^(39)^.

However, a video, also as an interactive proposal, recognized that participants with lower levels of education may require additional approaches^(23)^. In addition, there is a need to consider cultural and linguistic barriers in educational interventions^(6,24)^. Thus, it is necessary to consider the health literacy of the population for which the intervention is intended, since different populations within the same context may have different levels of understanding of health information^(43)^.

In terms of the methodology for constructing the intervention, some studies highlighted the use of theoretical references to support its structure and purpose, from theories focused on learning^(26)^ to theories that predispose to planned behavior change^(36)^. It has been found that the use of persuasive communication, derived from a theoretical model, is an effective strategy in technologies that propose the dissemination of knowledge^(6)^. The support of these elements contributes to the designed intervention being consistent with its purpose, including elements considered indispensable for the intended objective and thus enhancing its effect^(44)^.

In addition to the use of theory, combining videos with other resources highlights the synergy between different educational approaches, such as discussion sessions^(30)^ and informative printed material^(28-29,36)^, which promotes the dissemination of information in a more attractive way, involving closer contact with participants, who will have a space available to clarify their doubts or material for reading and further study whenever necessary.

Another point to consider is the positive impact of audiovisual interventions on STIs. Several studies have shown that videos, especially interactive ones, in addition to increasing knowledge, have contributed to a reduction in the acquisition of some STIs^(19)^. In addition, there is also the possibility of improving participants’ willingness to undergo STI/HIV screening tests^(25)^ and adopt preventive measures such as HPV vaccination^(36)^. Since these technologies have the potential to capture the public’s interest and facilitate the learning process, they improve the dissemination of health information and, consequently, lead to more frequent adoption of preventive actions^(22)^.

The effectiveness of interventions is also evident in specific populations, such as injecting drug users^(37)^, men who have sex with men^(21)^, men with intellectual disabilities^(26)^, and health professionals^(31)^. It is well known that people in vulnerable situations are at increased risk for STIs; therefore, health education and prevention actions for these populations are necessary. However, it is also recognized that it is important to ensure that these actions are offered on an ongoing basis, since knowledge can be changeable, depending on the life and health situation of certain audiences^(26)^.

When compared to face-to-face sessions, videos proved to be acceptable and not inferior in terms of helping participants understand the knowledge conveyed^(21,27-28)^. This demonstrates that, once applied to the context in which they are presented, they can lead to the same benefits as a lecture session alone, but with greater potential for reproduction and consequent dissemination to a larger number of people.

Among the limitations pointed out in the included studies, there is a need to consider the reliability of knowledge assessment instruments^(33)^, sample size, lack of specific clinical indicators (with prior diagnosis), and the need to consider cultural and linguistic barriers when implementing educational interventions^(6,23-24)^. In the context of HIV, as the most studied STI, it is also important to address the stigma related to infection within interventions^(31)^.

The results of this systematic review strengthen and support the use of audiovisual technologies in the dissemination of knowledge about STIs, to promote understanding, increase the level of information, and contribute to health promotion and prevention among young people and adults. None of the studies addressed the application of the intervention for older adults or people who need some form of accessibility (low vision or hearing); this is a gap that can be filled with new studies.

The applicability in healthcare practice lies in the ease of handling and use of these interventions, in addition to the potential for them to be accessed individually or collectively, favoring the dissemination of information in a broader and more effective way, as well as changing behaviors and attitudes towards STIs.

As this study did not have a time limit for the selection of studies, it is important to recognize that, over time, there have been significant changes in the social, cultural, and technological context that may influence the interpretation of the results. For example, the expansion of the Internet and digital devices has transformed the way people access information. In addition, there have been advances in the approach to STIs, which may have impacted risk perception and adherence to preventive measures over time.

However, it is important to highlight that audiovisual resources have proven to be effective methods for increasing knowledge about STIs and HIV/Aids at different times. A study included in this review, conducted in 2005^(34)^, as well as a doctoral thesis conducted in 2014^(45)^, were excluded for not meeting the eligibility criteria, as they used the same intervention with the same population, produced similar results, with significant increases in knowledge. This suggests that, despite contextual changes, well-structured interventions based on audiovisual resources can maintain their effectiveness over time.

Regarding limitations, the variability in the methodological quality of the included studies may have influenced the results, since many presented a high risk of bias. In addition, the exclusion of inaccessible studies and gray literature may have introduced a limitation in the scope of the research, which may not reflect all available evidence on the topic. It is possible that the exclusion of these materials resulted in the omission of relevant evidence, especially in contexts where published studies are scarce on the topic.

Another limitation concerns the different types of interventions analyzed, which may hinder the generalization of findings. In addition, the narrative synthesis of results, without a consolidated statistical analysis, may limit the accurate assessment of the effects of interventions, highlighting the need for new studies that are more homogeneous in terms of method.

## Conclusion

Audiovisual educational technologies, explained through videos, are effective in improving knowledge, attitudes, and practices regarding STIs and HIV/Aids. When adopting this type of resource, one must consider the socio-educational context in which the target audience is inserted, as well as cultural, linguistic, and health literacy aspects. Even without significant evidence, it is suggested that interventions based on theoretical models may be much more successful.

Since the magnitude of changes in knowledge can vary, it is necessary to consider the characteristics of the participants before designing or choosing the intervention to be applied, so that the essential elements of communication with the audience are present in the technology. Thus, knowledge retention and long-term changes in health behaviors should be considered to ensure that the information is perpetuated in the daily lives of those who received the intervention, while they also become multipliers of this knowledge.

## Data Availability

All data generated or analysed during this study are included in this published article.
