# Supplementary material for: Effectiveness of audiovisual interventions in increasing knowledge about sexually transmitted infections: a systematic review
Source: Rev Lat Am Enfermagem. 2025 Nov 10;33:e4704. doi: 10.1590/1518-8345.7531.4704 (PMC12610925; doi:10.1590/1518-8345.7531.4704)
Supplement: Translation

# Translation: Efetividade de intervenções audiovisuais no aumento do conhecimento sobre infecções sexualmente transmissíveis: uma revisão sistemática

**DOI:** 10.1590/1518-8345.7531.4705

**Authors:** Emanoelle Fernandes Silva, Polyana Norberta Mendes, Marli Teresinha Gimeniz Galvão, Danielle Nedson Rodrigues de Macêdo, Breno Dias de Oliveira Martins, Rosilane de Lima Brito Magalhães

**Affiliations:** 1 Universidade Federal do Piauí, Teresina, PI, Brasil. Universidade Federal do Piauí PI Teresina Brasil; 2 Bolsista do Conselho Nacional de Desenvolvimento Científico e Tecnológico (CNPq), Brasil. Bolsista do Conselho Nacional de Desenvolvimento Científico e Tecnológico Brasil; 3 Centro Universitário Santo Agostinho, Teresina, PI, Brasil. Centro Universitário Santo Agostinho PI Teresina Brasil; 4 Universidade Federal do Ceará, Fortaleza, CE, Brasil. Universidade Federal do Ceará CE Fortaleza Brasil; 5 Universidade Federal do Piauí, Departamento de Enfermagem, Teresina, PI, Brasil. Universidade Federal do Piauí Departamento de Enfermagem PI Teresina Brasil; 6 Bolsista da Universidade Federal do Piauí (UFPI), Brasil. Bolsista da Universidade Federal do Piauí Brasil

**Keywords:** Adolescente, Adulto, Idoso, Recursos Audiovisuais, Mídia Audiovisual, Infecções Sexualmente Transmissíveis

## Abstract

analisar as evidências sobre a efetividade de intervenções audiovisuais no aumento do conhecimento sobre Infecções Sexualmente Transmissíveis em diferentes faixas etárias.

revisão sistemática, realizada em 21 de fevereiro de 2023, na *Cumulative Index to Nursing and Allied Health Literature*, *Cochrane*, Embase, Literatura Latino-Americana e do Caribe em Ciências da Saúde, Base de Dados em Enfermagem, *Índice Bibliográfico Español en Ciencias de la Salud*, MEDLINE, *Scopus* e *Web of Science*. Incluíram-se estudos experimentais que utilizaram recursos audiovisuais para melhorar o conhecimento, comparados com outras intervenções ou nenhuma. A síntese dos resultados foi narrativa, com avaliação do risco de viés e certeza das evidências.

a amostra foi de 25 estudos (10 ensaios clínicos randomizados e 15 estudos quase-experimentais). O vídeo educativo foi o recurso mais utilizado. Em comparação com outra intervenção ou nenhuma, o grupo intervenção alcançou melhorias significativas no conhecimento a médio e longo prazo, além de mudanças na percepção e na predisposição para a redução de comportamento de risco. A certeza das evidências foi baixa para os estudos randomizados e muito baixa para os quase-experimentais.

as intervenções audiovisuais mostraram efetividade em diferentes populações e faixas etárias, destacando a necessidade de considerar parâmetros específicos na criação desses recursos.

## Destaques

**(1)** Eficácia das intervenções audiovisuais na melhoria do conhecimento sobre IST. **(2)** Mudanças de atitude e redução de comportamentos de risco após o uso da intervenção. **(3)** Capacidade de usabilidade das intervenções para as diferentes populações. **(4)** Recursos audiovisuais são aceitos como ferramenta de promoção e prevenção da saúde. **(5)** Uso de modelos teóricos para a construção das intervenções.

## Introdução

As Infecções Sexualmente Transmissíveis (IST) têm despertado, ao redor do mundo, um grande interesse para seu combate e enfrentamento. Esforços internacionais têm sido traçados a fim de alcançar taxas inferiores de incidência de diversas ISTs, como a sífilis, gonorreia, hepatites e o HIV/Aids^(1)^. Entre eles, destaca-se o investimento em ações inovadoras de prevenção e conscientização como, por exemplo, a oferta de educação em saúde para diferentes populações.

Evidencia-se que há uma baixa literacia em saúde relacionada a um alto comportamento de risco para IST na população em geral^(2)^, o que contribui para uma menor percepção de risco. Por outro lado, quando se eleva o nível de alfabetização em saúde, há um aumento na predisposição para a adoção de comportamentos preventivos, como, por exemplo, o uso do preservativo^(3)^, e como efeito, a redução dos casos de IST.

No contexto da educação em saúde, é notório que a modalidade Tecnologia Educacional (TE), vem ganhando espaço como um recurso para o aumento do conhecimento em saúde, com destaque para os recursos audiovisuais. Como o próprio nome sugere, estes envolvem o uso combinado de imagem e som. Exemplos incluem vídeos, apresentações de *slides*, animações, realidade virtual, transmissões ao vivo, holografia, multimídia interativa, entre outros^(4)^.

Quando aplicado no contexto da educação em saúde, esse tipo de intervenção tem proporcionado significativos efeitos na melhora do conhecimento e percepção de saúde^(5)^. Esses resultados também são coerentes quando aplicados, especificamente, no contexto das ISTs^(6)^, demonstrando que há evidências da contribuição de recursos audiovisuais na oferta da educação em saúde sexual.

Diante disso, verifica-se que o consumo de vídeos no contexto da saúde sobre IST teve um aumento nos últimos anos entre os adultos, principalmente por meio das redes sociais, ao passo que favoreceu a conscientização e a divulgação de conhecimento de forma efetiva^(7)^. À medida que a tecnologia audiovisual se torna cada vez mais presente no cotidiano, é crucial entender como essas ferramentas têm sido aplicadas para a educação em saúde, bem como a qualidade das intervenções e seus efeitos.

Desse modo, esta revisão tem por objetivo analisar evidências sobre a efetividade de intervenções audiovisuais no aumento do conhecimento sobre as ISTs em diferentes faixas etárias.

Espera-se que seja possível obter *insights* valiosos para os profissionais de saúde, educadores e formuladores de intervenções, a fim de contribuir com a construção de estratégias mais eficientes e adaptadas às necessidades contemporâneas de prevenção e promoção da saúde sexual.

## Método

### Delineamento do estudo

Esta revisão sistemática de efetividade foi realizada de acordo com as etapas metodológicas elaboradas pelo JBI^(8)^ e relatada conforme o *Reporting Items for Systematic Reviews and Meta-Analyses* (PRISMA)^(9)^. Este estudo está registrado no PROSPERO com o ID CRD42022374619.

### Modificações no protocolo *a priori*

Esta revisão foi realizada com as seguintes modificações no protocolo *a priori*:

- Não foram utilizados os instrumentos padronizados da JBI para a avaliação do risco de viés, optou-se por utilizar as ferramentas *Risk of Bias 2* (RoB 2) e *Risk Of Bias In Non-randomized Studies - of Interventions* (ROBINS-I) da *Cochrane*. Essa decisão foi tomada com base no amplo reconhecimento da sua avaliação em estudos randomizados e não randomizados. Como a revisão incluiu ambos, essas ferramentas foram consideradas as mais apropriadas para uma avaliação precisa e consistente. Além de serem amplamente utilizadas na literatura, são complementares aos princípios do método JBI e foram aplicadas de forma rigorosa e transparente, não comprometendo a integridade metodológica da revisão;

- Incluiu-se além do desfecho primário conhecimento, quando presente, os desfechos secundários atitudes e comportamentos;

- Os dados foram extraídos por meio de um instrumento criado pelos autores, com base nas características dos estudos incluídos;

- Apenas Ensaios Controlados Randomizados (ECRs) e estudos quase-experimentais foram incluídos. Anteriormente previu-se a inclusão de estudos de séries temporais e delineamentos observacionais analíticos. No entanto, durante o processo de seleção e avaliação dos estudos, observou-se que os ECRs e ensaios não randomizados atenderiam melhor ao objetivo do estudo, possibilitando uma avaliação mais direta e robusta da efetividade de intervenções, pois fazem comparações com grupos pré-estabelecidos, minimizando vieses e confundimentos. Ademais, a inclusão de estudos com delineamentos muito heterogêneos poderia comprometer a consistência dos resultados e a síntese das evidências.

### Pergunta de revisão

A pergunta foi formulada segundo a estratégia PICO, com P (População: jovens, adultos e idosos); I (Intervenção: recursos audiovisuais); C (Comparação: outras intervenções); O (*Outcomes*: conhecimento sobre IST). Dessa forma, a seguinte questão foi elaborada: qual é a efetividade de intervenções audiovisuais em comparação com outros tipos de intervenções no aumento do conhecimento sobre IST em jovens, adultos e idosos?

### Critérios de elegibilidade

Incluíram-se ECRs e estudos quase-experimentais. Não se realizou buscas diretas na literatura cinzenta. Dessa forma, ela foi excluída devido à variabilidade na qualidade metodológica e à falta de revisão por pares formal, que podem comprometer a confiabilidade das evidências.

A população abrangeu pessoas jovens, adultas e idosas^(10)^. É importante destacar que nem todos os artigos incluídos optaram por faixas etárias específicas, mas enfatiza-se que não ocorreu qualquer tipo de exclusão com base em questões etárias. Além disso, não houve restrição quanto ao sexo, escolaridade, renda, raça ou condição de saúde.

No que diz respeito ao tipo de intervenção, foram consideradas apenas aquelas que utilizaram recursos audiovisuais, ou seja, estruturadas com o uso de som e imagens associados. Isso incluiu vídeos educativos, programas multimídia, filmes documentários, curtas-metragens e DVDs interativos. Intervenções compostas apenas por chamadas de áudio sem vídeo, como no caso das fotonovelas ou radionovelas, foram excluídas.

Para a comparação do efeito da intervenção principal, foi considerado qualquer outro tipo de recurso, independentemente de ser ou não apoiado em mídia audiovisual.

O desfecho principal desta revisão foi o nível de conhecimento sobre ISTs, medido antes e depois da intervenção (avaliado por instrumentos estruturados). Os desfechos secundários foram aqueles relacionados às mudanças de comportamento (ações físicas ou práticas que contribuem para a prevenção) e atitude (posturas mentais ou emocionais) frente às ISTs (medidos por escalas ou relatos autorreferidos).

Não houve delimitação por idioma ou ano de publicação para os estudos.

### Fontes de informação e estratégia de busca

Para verificar a adequabilidade da pergunta de revisão, bem como a existência de revisões concluídas ou andamento sobre a temática, foi realizada uma busca inicial no PROSPERO, *JBI Evidence Synthesis* e na MEDLINE (*PubMed*). Não sendo encontradas revisões sistemáticas sobre o tema, procedeu-se à busca pelos termos de indexação principais no *Medical Subject Headings* (MeSH) e Descritores em Ciências da Saúde (DeCS).

As bases de dados pesquisadas foram a *Medical Literature Analysis and Retrieval System on-line* (MEDLINE via *PubMed*), *Scopus* (via *Elsevier*), *Web of Science* (via *Clarivate Analytics*), *Cumulative Index to Nursing and Allied Health Literature* (CINAHL via EBSCOhost), Literatura Latino-Americana e do Caribe em Ciências da Saúde (LILACS via BVS), *Índice Bibliográfico Español en Ciencias de la Salud* (IBECS via BVS), Base de dados de Enfermagem (BDENF via BVS), *Cochrane* (via *Wiley*) e *Embase* (via *Elsevier*).

A estratégia foi desenvolvida inicialmente na MEDLINE e posteriormente adaptada para cada base de dados, de acordo com suas especificações e/ou descritores específicos. A estratégia de busca foi então estruturada utilizando-se uma combinação de termos adequados, agrupados por categorias: população (jovens, adultos e idosos), intervenções (audiovisuais) e desfecho (ISTs). Foi realizada uma busca inicial em 11 de janeiro e uma final (atualização) em 21 de fevereiro de 2023.

Dessa forma, a expressão de busca adotada foi a seguinte: *# 1”Adolescent”[Mesh] OR Adolescent OR “Young Adult”[Mesh] OR (Young Adult) OR “Adult”[Mesh] OR Adult OR “Aged”[Mesh] OR Aged OR Adolescents OR Adolescence OR Teens OR Teen OR Teenagers OR Teenager OR Youth OR Youths OR (Adolescents, Female) OR (Adolescent, Female) OR (Female Adolescent) OR (Female Adolescents) OR (Adolescents, Male) OR (Adolescent, Male) OR (Male Adolescent) OR (Male Adolescents) OR (Adult, Young) OR (Adults, Young) OR (Young Adults) OR Adults OR Elderly AND #2 “Audiovisual Aids”[Mesh] OR (Audiovisual Aids) OR “Video-Audio Media” [Publication Type] OR (Video-Audio Media) OR “Webcast” [Publication Type] OR Webcast OR (Aid, Audiovisual) OR (Aids, Audiovisual) OR (Audiovisual Aid) OR (Audio-Visual Aids) OR (Aid, Audio-Visual) OR (Aids, Audio-Visual) OR (Audio Visual Aids) OR (Audio-Visual Aid) OR (Visual Aids) OR (Aid, Visual) OR (Aids, Visual) OR (Visual Aid) OR (Audiovisual Media) OR (Audio-Visual Media) OR Webcasts OR (Streaming Video) AND #3 “Sexually Transmitted Diseases”[Mesh] OR (Sexually Transmitted Diseases) OR “HIV”[Mesh] OR (HIV) OR “Syphilis”[Mesh] OR Syphilis OR “Hepatitis B”[Mesh] OR (Hepatitis B) OR “Chlamydia”[Mesh] OR Chlamydia OR “Gonorrhea”[Mesh] OR Gonorrhea OR “Trichomonas Infections”[Mesh] OR (Trichomonas Infections) OR “Herpes Genitalis”[Mesh] OR (Herpes Genitalis) OR “Papillomavirus Infections”[Mesh] OR (Papillomavirus Infections) OR (Disease, Sexually Transmitted) OR (Venereal Diseases) OR STDs OR (Sexually Transmitted Infections) OR (Infection, Sexually Transmitted) OR STIs OR STI OR (Human Immunodeficiency Virus) OR (AIDS Virus) OR (Great Pox) OR (Hepatitis B Virus Infection).*

As buscas e o acesso às bases foram realizados por dois revisores, utilizando acesso remoto institucional da Universidade Federal do Piauí (Teresina, Piauí, Brasil) por meio do Portal de Periódicos da Coordenação de Aperfeiçoamento de Pessoal de Nível Superior (CAPES). A lista final de referências dos artigos incluídos foi consultada e os autores correspondentes dos artigos que não estavam disponíveis na íntegra foram contatados, quando um meio de comunicação foi identificado.

### Seleção de estudos

Os estudos recuperados foram exportados para o *EndNote* (*Clarivate Analytics*, PA, USA) para a exclusão de duplicatas. Em seguida, os dados foram transferidos para o *software Rayyan*, onde ocorreu a etapa de seleção por meio da leitura de títulos e resumos, bem como a aplicação dos critérios de elegibilidade.

Dois revisores independentes, acompanhados por um terceiro, participaram do processo de triagem de forma cega. Os artigos elegíveis foram separados para leitura na íntegra, seguida pela reaplicação dos critérios de seleção. Aqueles que não atenderam aos requisitos pré-estabelecidos foram excluídos, e os motivos foram registrados. Os resultados do processo de seleção e inclusão são apresentados integralmente por meio do fluxograma disponível no PRISMA^(9)^, na seção Resultados.

### Coleta de dados, variáveis e medidas de efeito

O processo de coleta de dados foi realizado de maneira estruturada, garantindo a integridade e a precisão das informações extraídas dos estudos incluídos na revisão. Essa etapa também foi conduzida por dois revisores independentes, que utilizaram um formulário de coleta de dados desenvolvido para essa finalidade. Após a extração inicial, os dados foram registrados em uma planilha organizada para permitir comparações e revisões. Quando houve dúvidas em relação à extração das informações entre os revisores, um terceiro revisor era contatado para os esclarecimentos devidos.

O formulário utilizado abordava as seguintes variáveis: autores, ano e local de publicação, tamanho da amostra, características dos participantes (idade, sexo, raça, escolaridade e condição de saúde, se houver), tipo de estudo, IST para a qual se destinava a intervenção, tipo de recurso audiovisual utilizado nos Grupos de Intervenção (GI) e Grupo de Controle (GC), conteúdo da intervenção, tipo de instrumento para a avaliação do pré e pós-teste, tempo de seguimento, medidas de resultado, medidas de efeito para dados contínuos e/ou dicotômicos (*odds ratio*, diferença média e Intervalo de Confiança - IC), significância estatística e outros indicadores de risco de viés (randomização, ocultação de alocação, cegamento, completude do relatório).

Ressalta-se que os estudos incluídos apresentaram as informações necessárias para preencher o instrumento de forma qualitativa. Nos casos de dados quantitativos essenciais para a avaliação do conhecimento, em alguns estudos, essa informação apareceu de forma generalizada, focando apenas na significância estatística. Contatou-se os autores para solicitar dados ausentes, porém sem retorno. Alguns estudos, devido ao tempo de publicação ou própria formatação do periódico, não disponibilizaram meios de contato com autor correspondente.

O efeito foi avaliado para cada desfecho, quando disponível, por meio da diferença média pós-intervenção final, *odds ratio*, significância estatística e IC.

### Avaliação do risco de viés

A avaliação do risco de viés foi conduzida tanto para os ensaios clínicos randomizados quanto para os estudos quase-experimentais, utilizando as ferramentas disponibilizadas pela *Cochrane*: *a Revised Cochrane Risk-of-Bias tool for Randomized Trials* (RoB 2)^(11)^ e a *Risk Of Bias In Non-randomized Studies - of Interventions* (ROBINS-I)^(12)^, respectivamente. A ROB 2 avalia cinco domínios, que são viés de alocação, viés de desempenho, viés de detecção, viés de relato e viés de seleção de relatórios. Já a ROBINS-I abrange sete domínios, que incluem viés de confusão, viés de seleção dos participantes, viés de classificação da intervenção, viés de desvio do protocolo, viés de coleta de dados, viés de falta de dados e viés de relato seletivo.

Esse processo foi realizado por dois revisores independentes e será detalhadamente apresentado na seção Resultados. Independentemente da qualidade metodológica, todos os estudos foram submetidos ao processo de síntese e extração de dados, sendo posteriormente incluídos nesta revisão.

### Síntese dos resultados

Devido à variabilidade e heterogeneidade nos artigos incluídos, assim como à ausência de dados quantitativos completos, não foi possível realizar uma metanálise. A maioria dos estudos relatou os resultados de forma qualitativa, sem fornecer valores numéricos suficientes que permitissem a combinação estatística dos dados.

Além disso, a falta de padronização nos desfechos, medidos por instrumentos de conhecimento diferentes, e a descrição incompleta das intervenções audiovisuais em alguns estudos limitaram a comparação entre os estudos. Assim, a síntese dos dados é apresentada de forma narrativa.

Dessa forma, para facilitar a análise comparativa dos resultados, os estudos foram agrupados de acordo com o tipo de estudo. Cada um foi organizado pelo tipo de intervenção audiovisual (vídeos educativos, programas multimídia, DVDs), instrumento de avaliação do conhecimento e os desfechos medidos, incluindo o aumento do conhecimento sobre ISTs e mudanças nas atitudes e comportamentos.

Adotou-se o *Grading of Recommendations Assessment, Development and Evaluation Working Group* (GRADE)^(13)^, por meio do *software GRADEpro*^(14)^, para a avaliação da certeza das evidências. A análise foi feita para cada desfecho analisado, de acordo com o tipo de estudo.

## Resultados

### Inclusão dos estudos

Neste estudo, a busca por evidências foi realizada por meio de consulta às bases de dados eletrônicas e, adicionalmente, por uma busca manual nas listas de referências dos estudos incluídos. No entanto, a busca manual não resultou na identificação de novos estudos elegíveis para a inclusão na revisão. Dessa forma, optou-se por adaptar o fluxograma PRISMA, excluindo a etapa de “Identificação de estudos por outros métodos”, uma vez que ela não gerou acréscimos ao processo de seleção (Figura 1).

As buscas nas bases de dados resultaram em 2759 estudos; desses, 371 foram removidos por duplicatas. Em seguida, 2388 artigos foram selecionados para a leitura de títulos e resumos. Mediante a aplicação dos critérios de seleção, a amostra final foi composta por 25 estudos, sendo 10 ECRs e 15 estudos quase-experimentais. O processo de seleção e os motivos de exclusão são detalhadamente apresentados na Figura 1.

**Figura 1-:** Fluxograma do processo de seleção dos estudos incluídos na revisão sistemática adaptado do *Preferred Reporting Items for Systematic Review and Meta-Analyses* (PRISMA) (n = 25). Teresina, PI, Brasil, 2024

Fonte: Adaptado^(9)^

Durante a leitura na íntegra, cinco estudos não puderam ser recuperados. Desses, dois foram retirados de circulação e três permaneceram inacessíveis na íntegra, mesmo após tentativas de contato com os autores correspondentes.

Além disso, a busca na LILACS recuperou uma tese de doutorado que abordava a mesma intervenção educativa apresentada em um dos artigos incluídos nesta revisão. No entanto, após uma avaliação criteriosa, verificou-se que a tese não havia sido publicada em um periódico revisado por pares. Diante disso, optou-se por excluí-la a fim de manter a consistência metodológica e os critérios de elegibilidade predefinidos. Detalhes adicionais sobre a tese e sua relação com o estudo publicado estão descritos na seção Discussão.

### Avaliação do risco de viés

A avaliação do risco de viés nos estudos apresentou variações, indicando diferentes níveis de certeza sobre a validade dos resultados obtidos. No caso dos ECRs, observou-se que três estudos apresentaram baixo risco de viés, enquanto sete apresentaram alto risco. Entre esses, um estudo demonstrou alto risco de viés devido ao processo de randomização (D1), a aleatoriedade não foi garantida; um segundo, devido a desvios das intervenções pretendidas (D2), já que os participantes estavam cientes da intervenção recebida; o terceiro, devido à falta de dados de resultados (D3), evidenciada pela significativa perda de participantes durante o seguimento. Além disso, sete estudos revelaram alto risco de viés na mensuração do desfecho (D4), pois os avaliadores dos resultados estavam cientes da intervenção aplicada aos GI e GC (Figura 2).

No que diz respeito aos estudos quase-experimentais, nove apresentaram risco de viés moderado, três apresentaram risco sério e dois apresentaram risco de viés crítico. Os domínios mais suscetíveis ao viés foram o D3: risco de viés na classificação das intervenções, abrangendo 12 artigos, e o D6: risco de viés na medição dos resultados, envolvendo 13 artigos. Entre as principais limitações dos estudos quase-experimentais incluídos, destacaram-se a dificuldade em mascarar o conhecimento do resultado, dada a natureza de intervenção educativa avaliada por testes de conhecimento, e ausência de GC, além do não acompanhamento completo dos participantes nos estudos com seguimento. Adicionalmente, observou-se o conhecimento da intervenção recebida e o potencial para o conhecimento dos avaliadores do resultado em relação ao GI (Figura 3).

**Figura 2-:** Avaliação do risco de viés dos ensaios clínicos controlados randomizados em cada domínio da ferramenta RoB 2 (n = 10). Teresina, PI, Brasil, 2024

Fonte: Imagem criada pelo programa Robvis^(15)^

**Figura 3-:** Avaliação do risco de viés dos estudos quase-experimentais em cada domínio da ferramenta ROBINS-I (n = 15). Teresina, PI, Brasil, 2024

Fonte: Imagem criada pelo programa Robvis^(15)^

### Cenário e ano dos estudos incluídos

Os estudos incluídos apresentaram variação no ano de publicação, sendo que o estudo mais antigo foi de 1988^(16)^, enquanto o mais recente foi de 2022^(6)^. Em relação aos locais de realização das pesquisas, 13 (52%) foram conduzidos nos Estados Unidos^(16-28)^, quatro (16%) na Indonésia^(29-32)^, um (4%) em cada um dos seguintes países: Rússia^(33)^, Peru^(34)^, Brasil^(35)^, Canadá^(36)^, Porto Rico^(37)^, Sérvia^(38)^, México^(6)^ e não especificado (estudo*online*)^(39)^.

### Participantes

Os 25 estudos analisados totalizam 6021 participantes, dos quais 2176 pertencem aos 10 ECRs^(6,16,19,21,23-25,27-28,37)^ e 3845 aos estudos quase-experimentais^(17-18,20,22,26,29-35,37-39)^.

Entre os participantes observaram-se variações significativas. Três estudos foram realizados com estudantes universitários^(17-18,37)^, enquanto oito estudos focaram em adolescentes, com faixa etária variando de ≤ 13 anos a 21 anos^(19,25,28,30,32-36)^, sendo que um deles abordou também os cuidadores^(28)^. Quatro estudos foram conduzidos com pessoas vivendo com alguma IST^(16,20,22,37)^, e um estudo foi desenvolvido com homens com deficiência intelectual leve a moderada^(26)^.

Também foram incluídos participantes adultos atendidos em um departamento de emergência^(21)^, homens que fazem sexo com homens^(23)^, latinos hispanófonos^(27)^, usuários de *Internet*^(39)^, soldados das Forças Armadas^(38)^, profissionais da saúde^(31)^ e jovens maiores de 18 anos^(6)^. Vale ressaltar que a maioria dos estudos abordou exclusivamente o sexo masculino^(16,23-24,26,38)^, ao passo que dois foram desenvolvidos com o sexo feminino^(19,29)^. Não foi identificado nenhum estudo conduzido exclusivamente com pessoas idosas; a idade mais avançada relatada no público geral foi de 70 anos^(20)^.

### Intervenções/comparadores

As intervenções aplicadas serão abordadas de acordo com o tipo de estudo. Entre os ECRs, a modalidade mais adotada no GI foi o uso de vídeos. Cinco deles apoiaram-se apenas no uso de vídeos educativos, focando na disponibilização de informações sobre a gonorreia (*It Just Happens Sometimes*)^(16)^, HIV/Aids (*Do you know about rapid HIV testing?)*^(21)^; vídeo educacional em espanhol sobre HIV/Aids)^(27)^, sífilis (*Syphilis and Men*)^(23-24)^ e HPV (*7 cosas que debes saber sobre el VPH!*)^(6)^. Um vídeo foi interativo sobre IST de forma geral^(19)^, proporcionando não apenas informações, mas também elementos que permitiam aos participantes realizarem ações.

Além disso, um vídeo concentrou-se no aspecto educacional e no aconselhamento pré-teste do HIV^(25)^. Outra intervenção combinou o uso de panfleto educativo e vídeo para tratar sobre o HPV e vacinação^(36)^. Por fim, houve uma intervenção que utilizou um DVD em conjunto com um livro de atividades^(28)^, proporcionando uma abordagem mais extensa. Esta cobriu diversos módulos para promover a comunicação, prevenção e habilidades interpessoais relacionadas ao HIV/Aids (*Work It Out Together*).

O tempo de duração das intervenções também variou, com a maioria concentrando-se em um intervalo inferior a 10 minutos. O menor tempo registrado foi de três minutos^(6)^, enquanto o maior atingiu uma hora^(19)^.

Entre os comparadores, GC, destaca-se que dois não receberam nenhuma intervenção^(6,16)^, um recebeu o cuidado-padrão de atendimento sem ser submetido a uma intervenção^(23)^, dois receberam materiais de leitura^(19,36)^, três foram submetidos a aconselhamentos presenciais^(21,25,27)^, um recebeu um *palm card* (cartão como parte da campanha de *marketing* social em resposta à epidemia de sífilis)^(24)^, e um foi exposto a um DVD de promoção da saúde geral e não possuía foco específico na redução de riscos relacionados ao comportamento sexual ou uso de substâncias^(28)^. A síntese dos ECRs por tipo de intervenção é abordada na Figura 4.

Nos estudos quase-experimentais, a modalidade de vídeo também foi a mais utilizada, abrangendo vídeos informativos, educacionais e do tipo documentário. Três intervenções basearam-se no uso de programas educacionais multimídia^(22,37)^, sendo um deles interativo e acessível para pessoas com deficiência intelectual^(26)^. Uma intervenção adotou o formato de filme, do tipo curta-metragem^(38)^, enquanto outra envolveu um programa governamental que utilizou audiovisual aplicado por meio de sessões de discussão sobre a temática do HIV/Aids^(30)^.

Houve grande variação em relação ao tempo de duração das intervenções, sendo que alguns estudos não forneceram descrição detalhada. O menor tempo registrado foi de 15 minutos^(39)^, enquanto o maior relatou uma duração de duas horas^(33)^. Em relação aos GC, nove estudos não incluíram um GC, quatro não aplicaram nenhuma intervenção no grupo de comparação e apenas três compararam a intervenção principal com outra (Tabela 2 Figura 5).

Poucos foram os estudos que descreveram o meio de transmissão pelo qual as intervenções foram disponibilizadas aos participantes, a forma como a intervenção estava de fato estruturada, assim como se eram acessíveis de forma física ou eletrônica e se continham elementos de acessibilidade para as populações com alguma deficiência. Mais informações sobre as intervenções estão disponíveis nas Figuras 4 e 5.

Os referenciais teóricos utilizados na intervenção relatados nos estudos incluíram: Teoria da aprendizagem social de Bandura^(26)^, *Health Belief Model* (HBM)^(16,22,37)^, *Social Cognitive Theory* (SCT)^(22,37)^, *Health media and marketing*^(24)^, modelo teórico de modelos mentais simbólicos e ensaio cognitivo^(19)^, metodologia construtivista baseada em problemas e no pensamento crítico de Albion e Gibson^(34)^, Teoria da promoção da saúde, Teoria do comportamento planejado e Teoria da crença de saúde^(36)^ e Modelo de informação, motivação e habilidades comportamentais de Fisher e Fisher^(6)^.

### Desfecho

Os estudos abordados apresentaram uma diversidade de ISTs, 14 estudos focaram no HIV/Aids^(17-18,20-21,25-27,29-32,33,35,38-39)^, enquanto dois estudos abordaram o HIV e as ISTs de forma associada^(28,34)^. Dois estudos trataram do vírus da hepatite C^(22,37)^, um estudo abordou as ISTs de forma geral^(19)^, um estudo abordou a gonorreia^(16)^, dois estudos trataram da sífilis^(23-24)^, e dois estudos trataram do HPV^(6,36)^. O HIV tem sido a IST mais explorada no contexto de intervenções educativas audiovisuais (Figuras 4 e 5).

Em relação ao desfecho primário, todos os estudos avaliaram o conhecimento por meio de instrumentos aplicados antes e depois da intervenção, e houve uma melhora significativa (p<0,05) dessa variável no pós-teste para os grupos de tratamento; entretanto, dois estudos, entre os randomizados controlados, não apresentaram mudanças significativas após a aplicação da intervenção^(6,27)^. E, entre os quase-experimentais, apesar de mudanças estatisticamente significativas nas pontuações de conhecimento, dois estudos avaliaram que a magnitude dessas mudanças foi modesta^(17-18)^ (Figuras 4 e 5).

Não houve descrição nos estudos sobre como o formato em que as intervenções foram criadas impactou na melhora do conhecimento. Apenas um estudo relatou que uma abordagem de apresentação mais científica mostrou resultados melhores na avaliação do conhecimento do que uma mais informal^(35)^.

Apenas 11 estudos trataram outros desfechos além do conhecimento. A maioria envolveu a intervenção em mudanças de atitudes, e destacaram-se melhorias na adesão ao teste para o HIV^(25)^, vacinação contra o HPV^(36)^, redução de comportamentos sexuais de risco^(18-19,28)^ e no uso de substâncias^(28)^. Houve uma melhora na autoeficácia percebida em relação à adesão ao tratamento^(20)^, melhoria nas habilidades relacionadas ao HIV/Aids e uso de preservativos^(26)^, aumento do comportamento de proteção contra o HIV^(29)^ e mudanças de atitude frente ao HIV/Aids^(31-32)^. Não foram encontradas alterações na predisposição para notificar os contatos sexuais sobre o diagnóstico de IST^(16)^. Apenas um estudo tratou as mudanças de comportamento, focadas na redução no compartilhamento de seringas e equipamentos de preparação de drogas^(37)^ (Figuras 4 e 5).

**Figura 4-:** Síntese dos ensaios clínicos controlados randomizados incluídos (n = 10). Teresina, PI, Brasil, 2024

**Autores, ano e loc** al	**Amostra/tempo de seguimento**	GI*/tipo de tecnologia educativa	**GC** ^†^ **/tipo de tecnologia**	**Instrumento de avaliação do conhecimento**	**Principais resultados**
Solomon, DeJong. 1988. EUA ^( 16 )^	n=175/14 dias	**Vídeo:** “ *It Just Happens Sometimes* ” (10 min; n=83) Tipo/IST ^‡^ : Vídeo educativo/Gonorreia Conteúdo: Personagens discutindo sintomas, tratamento e prevenção, com ênfase em aconselhamento para o diagnóstico. Público-alvo: Homens negros com gonorreia.	Grupo sem vídeo educativo (n=92)	Teste verdadeiro e falso de 18 itens.	**Conhecimento:** Média de acertos GI* 16,6 (dp ^§^ = 1,7), GC ^†^ 13,3 (dp ^§^ = 2,2; 1(239) =13,4, *p* ^||^ <0,0001). **Atitude:** Disposição para notificar contatos sexuais ( *p* ^||^ >0,05) entre GI* e GC ^†^ .
Downs, et al. 2004. EUA ^( 19 )^	n=300/6 meses	**Vídeo:** (1 h; n=não explícito) Tipo/IST ^‡^ : Vídeo interativo/IST ^‡^ Conteúdo: Comportamentos de negociação, eficácia do uso de preservativos, anatomia reprodutiva feminina e respostas fisiológicas a infecções. Público-alvo: Adolescentes do sexo feminino.	Materiais de leitura (n=não explícito)	Teste verdadeiro e falso de 40 itens.	**Conhecimento:** Diferença média entre GI* e GC ^†^ , 5,3 pontos e 5,1 pontos, respectivamente. Melhorias significativas no conhecimento ao longo do tempo (geral: F ^¶^ (1,214= 69,09, *p* ^||^ <0,001; específico: F ^¶^ (1,214)= 58,62, *p* ^||^ <0,001).
Merchant, et al. 2009. EUA ^( 21 )^	n=574/não teve seguimento	**Vídeo:** “ Do you know about rapid HIV** testing ?” (9,5 min; n=270) Tipo/IST ^‡^ : Vídeo educativo/HIV** Conteúdo: Definição, transmissão, prevenção e testagem. Público-alvo: Adultos que frequentavam uma urgência e emergência.	Sessão presencial com conselheiro de teste do HIV** (n=304)	Questionário “Rapid HIV** Pretest Information Comprehension ” de 26 perguntas.	**Conhecimento:** Diferença média GI* vs. GC ^†^ **=** 0,68 (IC ^††^ 95%; 0,18-1,26; *p* ^||^ <0,05). As pontuações médias diferiram por menos de uma pergunta respondida corretamente.
Sánchez, et al. 2010. EUA ^( 23 )^	n=206/não teve seguimento	**Vídeo:** “ *Syphilis and Men* ” (5 min; n=100) Tipo/IST ^‡^ : Vídeo educativo/Sífilis Conteúdo: Transmissão, sintomas, tratamento, conexão entre sífilis-HIV** e redução de risco. Público-alvo: Homens que frequentavam uma urgência e emergência.	Cuidado-padrão sem vídeo sobre sífilis (n=106)	Questionário 10 questões de múltipla escolha.	**Conhecimento:** GI* pontuou em média 24,8% mais alto que GC ^†^ no teste pós-intervenção ( *p* ^||^ < 0,001).
Sánchez, Guilliames, et al. 2010. EUA ^( 24 )^	n=168/não teve seguimento	**Vídeo:** “ *Syphilis and Men* ” (5 min; n=85) Tipo/IST ^‡^ : Vídeo educativo/Sífilis Conteúdo: Transmissão, sintomas, tratamento, conexão entre sífilis-HIV** e redução de risco. Público-alvo: Homens negros que fazem sexo com homens.	*“Syphilis is Back” Palm Card* (n=83).	Questionário desenvolvido pelos pesquisadores (10 questões de múltipla escolha).	**Conhecimento:** GI* 8,9 a 9,8 perguntas corretas, GC ^†^ 6,9 a 7,9 perguntas corretas. Aumento de 19,5% para 20,9% na pontuação do pós-teste para GI* ( *p* ^||^ <0,05).
Calderon, et al. 2011. EUA ^( 25 )^	n=200/não teve seguimento	**Vídeo:** (4 min, n=100) Tipo/IST ^‡^ : Vídeo educativo/HIV** Conteúdo: Abrange sete elementos essenciais para uma sessão de aconselhamento pré-teste para HIV**. Público-alvo: Adolescentes.	Sessão presencial com conselheiro de teste do HIV** (n=100).	Questionário de 10 questões criado por Carey e Schroder, e questões do Departamento de Saúde do Estado de Nova York.	**Conhecimento:** Diferença média GI* vs. GC ^†^ = 12,2% ( *p* ^||^ <0,01); GI* 78,5% corretas e GC ^†^ 66,3% corretas. **Atitude:** Aceitar testagem para o HIV**: GI*, OR ^‡‡^ = 3,6x (IC ^††^ 95%:1,8–7,2; *p* ^||^ <0,001) comparado com GC ^†^ .
Krawczyk, et al. 2012. Canadá ^( 36 )^	n=200/não teve seguimento	**Vídeo** (5 min, n=74) **+ Panfleto educacional** (n=61) Tipo/IST ^‡^ : Vídeo + Panfleto/HPV ^§§^ Conteúdo: O vídeo apresenta um médico discutindo incidência, transmissão, consequências do vírus e eficácia da vacina. O panfleto traz informações sobre HPV ^§§^ e vacina. Público-alvo: Estudantes universitários.	Apenas panfleto sobre a prevenção do câncer de forma geral (n=65)	Teste de 22 itens (avaliação: verdadeiro, falso e não sei).	**Conhecimento:** Média de acertos GI* (panfleto)= 17,46; GI*(vídeo)= 16,70; GC ^†^ = 12,06 ( *p* ^||^ <0,001). **Atitude:** Aumento na intenção de vacinação para GI* (panfleto) e GI* (vídeo) em comparação com GC ^†^ ( *p* ^||^ < 0,001).
Merchant, et al. 2014. EUA ^( 27 )^	n=150/não teve seguimento	**Vídeo:** (9,5 min, n=75) Tipo/IST ^‡^ : Vídeo educativo/HIV** Conteúdo: Definições, natureza, transmissão, prevenção, métodos de teste e interpretação de resultados de HIV**. Público-alvo: Latinos hispanófonos.	Informação oral do conselheiro de teste de HIV** (n=75)	Teste de 25 itens (Avaliação: sim, não, não sei).	**Conhecimento:** Diferença média GI* vs. GC ^†^ = GI* 20,4 (IC ^††^ 95%: 19,5-21,3); GC ^†^ 20,6 (IC ^††^ 95%: 19,7-21,5), indicando semelhança (IC ^††^ 95%:-1,4-1,1).
Hadley, et al. 2016. EUA ^( 28 )^	n=170/3 meses	**DVD + livro** : “ *Work It Out Together* ” (20 min/módulo; n=83) Tipo/IST ^‡^ : DVD interativo + livro de atividades/HIV** Conteúdo: Seis módulos abordando comunicação entre pais e adolescentes, prevenção, uso de preservativos, negociação de sexo seguro, habilidades e tomada de decisões. Público-alvo: Adolescentes.	DVD de promoção da saúde geral (n=87)	Teste de conhecimento com 10 itens (verdadeiro, falso, incerto).	**Conhecimento:** Média de acertos GI*= 6,71; GC ^†^ = 6,45. Pais pontuaram mais (t ^||||^ (158)=2,00, *p* ^||^ <0,05). **Atitude:** GI* (pais) maior autoeficácia para se engajar em comportamentos de prevenção (t ^||||^ (158)= 2,28, *p* ^||^ <0,05). **Atitude:** GI*(adolescentes) maior autoeficácia para usar preservativos (t ^||||^ (64)= 2,06, *p* ^||^ <0,05).
Ortíz, et al. 2022. México ^( 6 )^	n=33/7 dias	**Vídeo:** “ *7 cosas que debes saber sobre el VPH§§!* ” (3 min; n=16) Tipo/IST ^‡^ : Vídeo educativo/HPV ^§§^ Conteúdo: Definição, formas de transmissão, sintomas, diagnóstico, tratamento e prevenção. Público-alvo: Jovens maiores de 18 anos.	Grupo sem vídeo educativo (n=17).	Teste de 38 itens (Verdadeiro, falso e não sei).	**Conhecimento:** GI* superior ao GC ^†^ ( *p* ^||^ =0,020 e *p* ^||^ =0,024, respectivamente). Média de acertos GI*= 30,75 (dp ^§^ = 2,32); GC ^†^ 28,18 (dp ^§^ = 3,55).

*GI = Grupo Intervenção; ^†^GC = Grupo Controle; ^‡^IST = Infecções Sexualmente Transmissíveis; ^§^dp = Desvio-Padrão; ^||^*p* = Significância Estatística; ^¶^F = Análise de Variância; **HIV = Vírus da Imunodeficiência Humana; ^††^IC = Intervalo de Confiança; ^‡‡^OR = *Odds Ratio*; ^§§^HPV = Papilomavírus Humano; ^||||^t = Estatística t (t de Student)

**Figura 5-:** Síntese dos estudos quase-experimentais incluídos (n = 15). Teresina, PI, Brasil, 2024

**Autores, ano e local**	**Amostra/tempo de seguimento**	GI*/tipo de tecnologia educativa	**GC** ^†^ **/tipo de tecnologia**	**Instrumento de avaliação do conhecimento**	**Principais resultados**
Rhodes, Wolitski. 1989. EUA ^( 17 )^	n=441/6 semanas	**Vídeos:** (16-20 min; n=não explícito) Tipo/IST ^‡^ : 4 Vídeos informativos/Aids Conteúdo: (1) *Aids: Acquired Immune Deficiency Syndrome,* 1986, 18 min; (2) Aids: *What Everyone Needs to Know* , 1986, 18 min; (3) *Beyond Fear: The Virus,* 1986, 20 min; (4) *Sex, Drugs, and Aids,* 1986, 16 min. Público-alvo: Estudantes universitários.	Não assistiram a nenhum dos vídeos (n= não explícito).	Teste verdadeiro e falso de 23 itens.	**Conhecimento:** Aumento no conhecimento nos quatro vídeos ( *p* ^§^ <0,001), superior ao GC ^†^ no pós-teste tardio ( *p* ^§^ <0,01).
Lipson, Brown. 1991. EUA ^( 18 )^	n=144/1 mês	**Vídeo:** (19-40 min; n=90) Tipo/IST ^‡^ : 3 Vídeos Informativos/Aids Conteúdo: (1) *Sex, drugs, and Aids* , 19 min; (2) *Aids: beyond fear* , 40 min; (3) *Aids: what you need to know* , 40 min Público-alvo: Estudantes universitários.	Videoteipe “ *Where Did I Come From* ?”, aborda a educação sexual, mas não o HIV ^||^ /Aids (n=54).	Questionário de 55 itens (A resposta mais correta recebeu nota 4, e a menos correta nota 0).	**Conhecimento:** Diferença média GI* vs. GC ^†^ = 15,93 pontos ( *p* ^§^ <0,01). **Atitude:** GI* melhorias nas atitudes em relação à comportamentos preventivos, diferença média de 1,33 pontos em relação GC ^†^ ( *p* ^§^ <0,01).
Torabi, et al. 2000. Rússia ^( 33 )^	n=1.124/2 semanas	**Vídeo:** (2 h; n=731) Tipo/IST ^‡^ : Vídeo informativo/HIV ^||^ Conteúdo: Prevenção, utilizou técnicas de palestra, ilustração e perguntas e respostas. Público-alvo: Adolescentes.	Programa acadêmico usual, excluindo a educação sobre HIV ^||^ /Aids (n=393).	Teste de 51 perguntas (“sim”, “não” e “não tenho certeza”).	**Conhecimento:** Pontuações do GI* em comparação ao GC ^†^ foram superiores (F ^¶^ =58,73, *p* ^§^ <0,01).
Velásquez. 2005. Peru ^( 34 )^	n=454/não teve acompanhamento	**CD:** “ *Planeta Riesgo Xero”* (duração não descrita; n=454) Tipo/IST ^‡^ : CD educativo/ IST ^‡^ e HIV ^||^ Conteúdo: Histórias cotidianas de adolescentes, jogo sobre a tomada de decisões, informações gerais, riscos, mitos e crenças. Público-alvo: Adolescentes.	Não teve GC.	Questionário de 101 perguntas dividido em quatro seções.	**Conhecimento:** Aumento de 3x mais no conhecimento sobre o HIV ^||^ e 6x mais sobre a Aids ( *p* ^§^ <0,001). Probabilidade aumentada de conhecer gonorreia (OR**=5,4), sífilis (OR**=4,8), clamídia (OR**=13,2), herpes genital (OR**=3,7) e cancro mole (OR**=2,3, *p* ^§^ <0,001).
Brock, Smith. 2007. EUA ^( 20 )^	n=51/4 a 6 semanas	**Vídeo:** (25 min; n=51) Tipo/IST ^‡^ : Vídeo educacional/HIV ^||^ Conteúdo: Exibido em um *Personal Digital Assistant* (PDA), contendo informações para a adesão à medicação antirretroviral. Público-alvo: Pacientes de ambulatório de doenças infecciosas.	Não teve GC.	Instrumento com nove perguntas	**Conhecimento:** Melhorias no conhecimento do HIV ^||^ e do tratamento ( *p* ^§^ <0,005). **Atitude:** Aumento na autoeficácia para a adesão ao tratamento ( *p* ^§^ <0,005).
Camargo, Barbará, Bertoldo. 2008. Brasil ^( 35 )^	n=141/1 semana	**Vídeos:** (duração não descrita) Tipo/IST ^‡^ : Vídeos documentários/HIV ^||^ Conteúdo: Vídeo 1, abordagem científica, desenvolvimento da Aids, prevenção e detalhes da doença (n=46). Vídeo 2, informações básicas e depoimentos de adolescentes vivendo com o vírus (n=39). Público-alvo: Adolescentes.	Não assistiram a nenhum dos vídeos (n=56).	Teste de conhecimento científico sobre a Aids.	**Conhecimento:** GI*1 apresentou melhor conhecimento em relação ao GI*2 e do GC ^†^ (F ^¶^ =8,15; *p* ^§^ <0,001).
Mayor. 2010. EUA ^( 22 )^	n=110/8 semanas	**Programa multimídia:** (25-35 min/sessão; n=110) Tipo/IST ^‡^ : Programa educacional/ HCV ^††^ Conteúdo: Desenvolvido no *PowerPoint* , aborda a suscetibilidade, medidas de proteção e relação com o HIV ^||^ . Público-alvo: Pacientes vivendo com HIV ^||^ .	Não teve GC.	Questionário autoaplicável com escala Likert.	**Conhecimento:** A compreensão de que a hepatite C é uma condição viral aumentou de 73,1% para 85,2% ( *p* ^§^ =0,03). Compreensão de que o HCV ^††^ é encontrada predominantemente no sangue aumentou de 92% para 98,2% ( *p* ^§^ =0,01).
Mayor. 2013. Porto Rico ^( 37 )^	n=88/6 meses	**Programa multimídia:** (duração não descrita; n=88) Tipo/IST ^‡^ : Programa educacional /HCV ^††^ Conteúdo: Desenvolvido no *PowerPoint* , aborda a suscetibilidade, medidas de proteção e relação com o HIV ^||^ . Público-alvo: Usuários de drogas com HIV ^||^ .	Não teve GC.	Questionário autoaplicável com escala Likert.	**Conhecimento:** Aumento na conscientização sobre manifestações clínicas, comportamentos de risco, práticas de prevenção e sinergismos de coinfecção HIV ^||^ /HCV ^††^ ( *p* ^§^ <0,05). **Comportamento:** Redução no compartilhamento de seringas e equipamentos de preparação de drogas de 39,8% para 13,6% ( *p* ^§^ <0,001).
Wells, Clark, Sarno. 2014. EUA ^( 26 )^	n=49/não teve seguimento	**Programa multimídia:** (1,5 h, n=49) Tipo/IST ^‡^ : Programa interativo/HIV ^||^ Conteúdo: Baseado em computador, focado na prevenção. Público-alvo: Homens com deficiência intelectual leve a moderada.	Não teve GC.	Questionário com 44 perguntas.	**Conhecimento:** Ganhos todas as áreas avaliadas, com um tamanho médio do efeito de 0,67 (efeito grande).
Jadranin. 2015. Sérvia ^( 38 )^	n=102/ não teve seguimento	**Filme:** *“HIV/Aids prevention and control in the Serbian Armed Forces”* (17 min, n=102) Tipo/IST ^‡^ : Filme educativo/HIV ^||^ Conteúdo: Prevenção, transmissão, progressão da doença e testagem. Público-alvo: Soldados das Forças Armadas.	Não teve GC.	Questionário de 23 perguntas de múltipla escolha.	**Conhecimento:** Diferença média antes e depois = 1,91 pontos, com aumento na pontuação após a intervenção ( *p* ^§^ <0,001).
Shao. 2016. Estudo *online* ^( 39 )^	n=817/6 semanas	**Vídeo:** (15 min, n=817) Tipo/IST ^‡^ : Vídeo informativo/HIV ^||^ Conteúdo: Utiliza animação para informar sobre a infecção aguda e os métodos de testagem. Público-alvo: Usuários da *Internet* .	Não teve GC.	Questionário de 25 perguntas + uma única pergunta em uma escala de 4 pontos.	**Conhecimento:** Diferença média antes e depois ao vídeo= falantes de inglês (Pós:19,6 vs. Pré:16,4; Δ ^‡‡^ =3,2; IC ^§§^ 95%: 2,8-3,5) e falantes de espanhol (Pós:20,7 vs. Pré:17,3; Δ ^‡‡^ =3,4; IC ^§§^ 95%: 3,0-3,8), com os falantes de inglês apresentando menor conhecimento prévio ( *p* ^§^ <0,05).
Dewi, Amir, Nauli. 2019. Indonésia ^( 29 )^	n=144/não teve seguimento	**Vídeo + folhetos:** (n=72) Tipo/IST ^‡^ : Vídeo educativo + folheto/HIV ^||^ Conteúdo: Informações para aumentar a compreensão e promover comportamentos de prevenção. Público-alvo: Mulheres casadas.	Não recebeu nenhuma das intervenções (n=72).	Questionário testado quanto à validade e confiabilidade	**Conhecimento:** Diferença média entre GI* vs. GC ^†^ = 3,1; GI*(12,86) e GC ^†^ (9,69) (IC ^§§^ 95%:2,60- 3,73, *p* ^§^ = 0,000). **Atitude:** Diferença média de 8,4 para atitude de prevenção entre GI* e GC ^†^ (IC ^§§^ 95%: 0,879-6,66, *p* ^§^ =0,000).
Nuramalia et al. 2020. Indonésia ^( 30 )^	n=96/4 semanas	**Mídia audiovisual ABAT:** *“I’m Proud I Know”* (120 min/sessão; n=48) Tipo/IST ^‡^ : Mídia educativa/HIV ^||^ Conteúdo: Saúde reprodutiva, drogas, estilo de vida, HIV ^||^ e Aids. Público-alvo: Adolescentes.	Uma única sessão por semana (duração de três semanas), abordando os mesmos temas (n=48).	Questionário com perguntas de conhecimento e práticas de prevenção.	**Conhecimento:** Aumento na categoria “Bom” para 91,6%, enquanto a categoria “Suficiente” foi de 8,3%. Houve diferenças no conhecimento entre o GI* e o GC ^†^ ( *p* ^§^ < 0,05).
Prihanti. 2020. Indonésia ^( 31 )^	n=41/não teve seguimento	**Vídeo:** (duração não descrita; n=41) Tipo/IST ^‡^ : Vídeo *online* /HIV ^||^ Conteúdo: Contém informações sobre a imagem geral do HIV, fatores de risco, transmissão e a importância do teste diagnóstico. Público-alvo: Profissionais de saúde.	Não teve controle.	*E-Questionnaire* via *Google Form* com 28 perguntas.	**Conhecimento:** 97,6% dos participantes demonstrando bom conhecimento e apenas 2,4% mantendo conhecimento insuficiente ( *p* ^§^ =0,00). **Atitude:** 85,4% dos participantes demonstraram atitudes positivas e 14,6% mantendo atitudes negativas ( *p* ^§^ =0,00).
Ambarwati. 2021. Indonésia ^( 32 )^	n=43/não teve seguimento	**Vídeo +** slides **:** (n=43) Tipo/IST ^‡^ : Vídeo educativo + *slides* /HIV ^||^ Conteúdo: Desenvolvidos para melhorar o conhecimento e atitudes em relação ao HIV ^||^ /Aids. Público-alvo: Adolescentes.	Não teve controle.	Questionário com perguntas não detalhados.	**Conhecimento:** Aumento no conhecimento (Z ^||||^ =-3,819; *p* ^§^ =0,000). **Atitude:** Aumento na atitude após a intervenção (Z ^||||^ =-3,873; *p* ^§^ =0,00).

*GI = Grupo Intervenção; ^†^GC = Grupo Controle; ^‡^IST = Infecções Sexualmente Transmissíveis; ^§^*p* = Significância Estatística; ^||^HIV = Vírus da Imunodeficiência Humana; ^¶^F = Análise de Variância; **OR = *Odds Ratio*; ^††^HCV = Vírus da Hepatite C; ^‡‡^Δ = Estatística Delta; ^§§^IC = Intervalo de Confiança; ^||||^Z = Estatística Z

A avaliação da certeza das evidências de acordo com os desfechos selecionados é apresentada na Figura 6. A qualidade das evidências foi classificada como baixa para ECRs e muito baixa para estudos quase-experimentais, devido a fatores como risco de viés grave ou extremamente sério, imprecisão (IC amplos ou falta de dados quantitativos) e inconsistência não grave. A maioria dos estudos apresentou limitações metodológicas, como alto risco de viés e imprecisão, o que impactou a confiabilidade dos resultados.

**Figura 6-:** Avaliação da certeza das evidências, segundo o *Grading of Recommendations Assessment, Development and Evaluation* (GRADE). Teresina, PI, Brasil, 2024

**Certeza da evidência**							**Número de pacientes**		**Efeito** ^††^		**Certeza**
**Nº dos estudos**	**Delineamento do estudo**	**Risco de viés**	**Inconsistência**	**Evidência indireta**	**Imprecisão**	**Outras considerações**	**Recursos audiovisuais**	**Nenhum ou outro**	Relativo (95% IC*)	Absoluto (95% IC*)	
**Aumento do conhecimento sobre IST**											
10	ECR ^†^	grave ^‡^	não grave	não grave	grave ^§^	nenhum	886/1815 (48,8%)	929/1815 (51,2%)	não estimável	não estimável	⨁⨁◯◯ Baixa ^‡§^
15	Estudos quase-experimentais	extremamente sério ^||^	não grave	não grave	grave ^§^	nenhum	2781/3404 (81,7%)	623/3404 (18,3%)	não estimável	não estimável	⨁◯◯◯ Muito baixa ^§||^
**Mudança de atitude**											
4	ECR ^†^	grave ^¶^	não grave	não grave	grave ^§^	nenhum	327/671 (48,7%)	344/671 (51,3%)	não estimável	não estimável	⨁⨁◯◯ Baixa ^§¶^
5	Estudos quase-experimentais	extremamente sério	não grave	não grave	grave ^§^	nenhum	297/423 (70,2%)	126/423 (29,8%)	não estimável	não estimável	⨁◯◯◯ Muito baixa ^§^ **
**Mudança de comportamento**											
1	Estudos quase-experimentais	extremamente sério	não grave	não grave	grave ^§^	nenhum	88/88 (100,0%)	0/0	não estimável	não estimável	⨁◯◯◯ Muito baixa ^§^ **

*IC = Intervalo de Confiança; ^†^ECR = Ensaio Clínico Randomizado; ^‡^Sete estudos apresentaram risco alto de viés de acordo com a ferramenta ROB-2; ^§^Alguns resultados apresentaram Intervalos de Confiança amplos, indicando imprecisão. Para outros resultados, há carência de dados quantitativos, o que impede a avaliação completa da precisão; ^||^Dois estudos apresentaram risco crítico de viés de acordo com a ferramenta ROBINS-I; ^¶^Três estudos apresentam risco alto de viés de acordo com a ferramenta ROB-2; **Um estudo apresentou risco crítico de viés de acordo com a ferramenta ROBINS-I, ^††^Não estimável devido à variabilidade dos estudos que impossibilitou a metanálise

## Discussão

O objetivo desta revisão sistemática foi analisar as evidências disponíveis sobre a efetividade de intervenções audiovisuais no aumento do conhecimento sobre IST em jovens, adultos e idosos. Com base nos resultados, foi possível evidenciar que esses recursos contribuem para uma homogeneidade do conhecimento factual, principalmente entre adolescentes e jovens-adultos. Essas populações são consideradas de risco para as IST, visto que na faixa etária de 13 a 34 anos concentram-se mais da metade (56%) de novos casos do HIV^(40)^. Logo, as intervenções têm sido focadas na parcela da população que mais necessita de acesso a informações sobre a temática.

A maioria dos estudos incluídos apontaram que intervenções educacionais audiovisuais têm sido amplamente exploradas como ferramentas eficazes na promoção do conhecimento sobre IST, especialmente o HIV/Aids. Além disso, há um impacto destas tecnologias na mudança de atitude e, em alguns casos, redução de comportamentos de risco relacionados a estas infecções.

Destaca-se que as tecnologias audiovisuais, por serem consideradas parte das Tecnologias da Informação e Comunicação (TIC), também se classificam como “tecnologias leve-duras”. Ou seja, elas se apresentam por meio de *hardwares* e dispositivos e necessitam de outros recursos, como câmeras, microfones, alto-falantes, monitores, além de *software* relacionado à produção e reprodução de conteúdo audiovisual^(41)^.

Esse tipo de tecnologia, “leve-dura”, tem potencial para despertar o aprendizado e a mudança de comportamentos em saúde, uma vez que possibilitam uma abordagem capaz de estimular os sentidos e manter os interessados mais firmemente atentos ao conteúdo que elas se propõem a apresentar^(42)^. Pode-se inferir que intervenções apoiadas nesse tipo de classificação podem ter efeitos satisfatórios no aumento do conhecimento em saúde e na adoção de práticas e comportamentos mais seguros.

Como descrito, houve um consenso entre as publicações a respeito do impacto do audiovisual no conhecimento. Em nível geral, é possível perceber que as informações repassadas foram bem absorvidas pelos participantes no pós-teste^(19)^. Somado a isso, também é destaque a influência de vídeos educacionais na melhora significativa do conhecimento sobre HIV/Aids, em comunidades norte-americanas onde o espanhol é dominante^(27)^.

Todavia, apesar do grande quantitativo de desfechos mostrando melhorias estatisticamente significativas no conhecimento, é preciso considerar também que a força dessas mudanças pode ser modesta^(17)^. À vista disso, deve-se considerar de forma inclusa a relevância clínica, para além da significância estatística. Em outras palavras, existe a necessidade de observar essa melhora do conhecimento juntamente com a predisposição dos participantes em reduzir os comportamentos de risco e adotar medidas de prevenção em relação às IST.

A diversidade de contextos nos quais os estudos foram realizados, incluindo diferentes populações (direta e indiretamente inseridas no contexto das IST), faixas etárias e níveis de alfabetização em saúde, destaca a flexibilidade e a adaptabilidade das intervenções com vídeos educacionais na promoção da saúde. Contudo, a generalização dos resultados para a população em geral pode ser limitada, especialmente quando a população-alvo está inserida no contexto escolar e possui níveis de alfabetização considerados satisfatórios para a idade que apresenta^(35)^.

Poucos foram os estudos que observaram se o nível de escolaridade dos participantes pode interferir no processo de aprendizado. Uma abordagem de vídeo dinâmico, como por exemplo, contando uma história baseada em personagens sobre a temática da gonorreia, no contexto de negociar com as parcerias a respeito da notificação e exames para IST, levou todos os pacientes do grupo de tratamento a um alto nível de conhecimento^(16)^, independentemente do nível educacional^(39)^.

No entanto, um vídeo, também como uma proposta interativa, reconheceu que participantes com menor escolaridade podem necessitar de abordagens adicionais^(23)^. Além disso, existe uma necessidade de se considerar barreiras culturais e linguísticas em intervenções educacionais^(6,24)^. Dessa forma, torna-se necessário considerar a literacia em saúde da população à qual se destina a intervenção, uma vez que diferentes populações dentro de um mesmo contexto podem apresentar níveis diferentes de compreensão de informações em saúde^(43)^.

Em termos de metodologia de construção da intervenção, alguns estudos destacaram o uso de referenciais teóricos para embasar a estrutura da mesma e sua finalidade, desde teorias voltadas à aprendizagem^(26)^, até teorias que predispõem às mudanças de comportamento de forma planejada^(36)^. Verifica-se que o uso de uma comunicação persuasiva, proveniente de um modelo teórico, é uma estratégia eficaz em tecnologias que propõem a divulgação de conhecimento^(6)^. O apoio desses elementos contribui para que a intervenção projetada seja coerente com sua finalidade, incluindo elementos considerados indispensáveis para o objetivo que se pretende atingir e dessa forma potencializando seu efeito^(44)^.

Além do uso de uma teoria, a combinação de vídeos com outros recursos ressalta a sinergia entre diferentes abordagens educacionais, como no caso de sessões de discussões^(30)^ e material impresso informativo^(28-29,36)^, que favorece uma divulgação de informações de forma mais atrativa, envolvendo um contato mais próximo com os participantes que terão à disposição um espaço para esclarecer suas dúvidas ou um material para leitura e aprofundamento sempre que necessário.

Outro ponto a ser considerado é o impacto positivo das intervenções audiovisuais nas ISTs. Diversos estudos demonstraram que os vídeos, principalmente os interativos, além de aumentarem o conhecimento, contribuíram para uma redução na aquisição de alguma IST^(19)^. Ademais, também existe a possibilidade de melhorar a disposição dos participantes a realizar os exames de triagem para IST/HIV^(25)^ e a adotar medidas de prevenção como a vacinação contra o HPV^(36)^. Uma vez que essas tecnologias têm potencial para captar o interesse do público e facilitar o processo de aprendizagem, elas melhoram a divulgação de informações em saúde e, por consequência, levam a uma adoção de ações preventivas mais assíduas^(22)^.

A resposta da eficácia das intervenções também é evidenciada em populações específicas, como os usuários de drogas injetáveis^(37)^, homens que fazem sexo com homens^(21)^, homens com deficiência intelectual^(26)^ e profissionais da saúde^(31)^. É de conhecimento que pessoas em alguma situação de vulnerabilidade apresentam um risco aumentado para IST, portanto, ações de educação em saúde e prevenção para essas populações se fazem necessárias. Todavia, também é reconhecida a importância de garantir que essas ações sejam ofertadas de forma contínua, visto que o conhecimento pode ser mutável, dependendo da situação de vida e saúde em que determinados públicos estão inseridos^(26)^.

Quando comparados às sessões presenciais, os vídeos se mostraram aceitáveis e não inferiores, no sentido de levar os participantes à compreensão do conhecimento repassado^(21,27-28)^. Isso demonstra que, uma vez aplicados ao contexto em que se apresentam, eles podem levar aos mesmos benefícios que uma sessão expositiva apenas, porém, com um potencial maior para a reprodução e consequente disseminação para um quantitativo maior de pessoas.

Entre as limitações apontadas nos estudos incluídos, é necessário considerar a confiabilidade dos instrumentos de avaliação do conhecimento^(33)^, tamanho da amostra, falta de indicadores clínicos específicos (com o diagnóstico prévio) e as barreiras culturais e linguísticas ao implementar intervenções educacionais^(6,23-24)^. No contexto do HIV, como sendo a IST mais trabalhada, há ainda a importância de abordar o estigma relacionado à infecção dentro das intervenções^(31)^.

Os resultados desta revisão sistemática fortalecem e apoiam o uso de tecnologias audiovisuais na disseminação de conhecimento sobre as IST, de modo a favorecer o entendimento, aumentando o nível de informação e contribuindo para a promoção e prevenção da saúde em jovens e adultos. Nenhum dos estudos abordou a aplicação da intervenção para pessoas idosas ou pessoas que necessitem de alguma acessibilidade (baixa visão ou audição); essa é uma lacuna que pode ser preenchida com novos estudos.

A aplicabilidade na prática assistencial está na facilidade de manuseio e uso dessas intervenções, além do potencial para serem acessadas de forma individual ou coletiva, favorecendo a disseminação de informações de forma mais ampliada e efetiva, bem como a mudança de comportamentos e atitudes frente às ISTs.

Como este estudo não possuiu limite temporal para a seleção dos estudos, é importante reconhecer que, ao longo do tempo, ocorreram mudanças significativas no contexto social, cultural e tecnológico que podem influenciar a interpretação dos resultados. Por exemplo, a ampliação da *Internet* e os dispositivos digitais transformaram a forma como as pessoas têm acesso às informações. Além disso, houve avanços relacionados à abordagem das ISTs, o que pode ter impactado a percepção de risco e a adesão às medidas preventivas ao longo do tempo.

No entanto, é importante destacar que os recursos audiovisuais demonstraram ser métodos eficazes para aumentar o conhecimento sobre ISTs e HIV/Aids em diferentes momentos. Tanto um estudo realizado em 2005 incluído nesta revisão^(34)^, quanto uma tese de doutorado realizada em 2014^(45)^, excluída por não atender aos critérios de elegibilidade, produziram resultados semelhantes ao utilizarem a mesma intervenção com a mesma população, com aumentos significativos no conhecimento. Isso sugere que, apesar das mudanças contextuais, intervenções bem estruturadas e baseadas em recursos audiovisuais podem manter sua eficácia ao longo do tempo.

Em relação às limitações, a variabilidade na qualidade metodológica dos estudos incluídos pode ter influenciado os resultados, uma vez que muitos apresentaram alto risco de viés. Além disso, a exclusão de estudos não acessíveis e da literatura cinzenta pode ter introduzido uma limitação na abrangência da pesquisa, podendo não refletir toda a evidência disponível sobre o tema. É possível que a exclusão desses materiais tenha resultado na omissão de evidências relevantes, especialmente em contextos em que há escassez de estudos publicados sobre o tema.

Outra limitação refere-se aos diferentes tipos de intervenções analisadas, que podem dificultar a generalização dos achados. Além disso, a síntese narrativa dos resultados, sem uma análise estatística consolidada, pode limitar a avaliação precisa dos efeitos das intervenções, destacando a necessidade de novos estudos mais homogêneos quanto ao método.

## Conclusão

As tecnologias educativas audiovisuais, elucidadas por meio de vídeos, são eficazes na melhoria do conhecimento, atitudes e práticas em relação às ISTs e HIV/Aids. Ao adotar esse tipo de recurso, deve-se considerar o contexto socioeducacional ao qual o público a que se destina está inserido, assim como os aspectos culturais, linguísticos e de alfabetização em saúde. Mesmo sem uma comprovação significativa, sugere-se que intervenções baseadas em modelos teóricos podem ser bem mais sucedidas.

Uma vez que a magnitude das mudanças no conhecimento pode variar, é preciso considerar as características dos participantes antes da construção ou escolha da intervenção a ser aplicada, a fim de que os elementos essenciais de comunicação com o público estejam presentes na tecnologia. Assim, deve-se considerar a retenção do conhecimento e das mudanças nos comportamentos de saúde a longo prazo, para garantir que as informações perpetuem no cotidiano daqueles que receberam a intervenção, ao passo que eles também se tornem multiplicadores desse conhecimento.

## Data Availability

Todos os dados gerados ou analisados durante este estudo estão incluídos neste artigo publicado.

# Translation: Efectividad de intervenciones audiovisuales en el aumento del conocimiento sobre infecciones sexualmente transmisibles: revisión sistemática

**DOI:** 10.1590/1518-8345.7531.4703

**Authors:** Emanoelle Fernandes Silva, Polyana Norberta Mendes, Marli Teresinha Gimeniz Galvão, Danielle Nedson Rodrigues de Macêdo, Breno Dias de Oliveira Martins, Rosilane de Lima Brito Magalhães

**Affiliations:** 1 Universidade Federal do Piauí, Teresina, PI, Brasil. Universidade Federal do Piauí PI Teresina Brasil; 2 Becaria del Conselho Nacional de Desenvolvimento Científico e Tecnológico (CNPq), Brasil. Becaria del Conselho Nacional de Desenvolvimento Científico e Tecnológico Brasil; 3 Centro Universitário Santo Agostinho, Teresina, PI, Brasil. Centro Universitário Santo Agostinho PI Teresina Brasil; 4 Universidade Federal do Ceará, Fortaleza, CE, Brasil. Universidade Federal do Ceará CE Fortaleza Brasil; 5 Universidade Federal do Piauí, Departamento de Enfermagem, Teresina, PI, Brasil. Universidade Federal do Piauí Departamento de Enfermagem PI Teresina Brasil; 6 Becario de la Universidade Federal do Piauí (UFPI), Brasil. Becario de la Universidade Federal do Piauí Brasil

**Keywords:** Adolescente, Adulto, Anciano, Recursos Audiovisuales, Medios Audiovisuales, Enfermedades de Transmisión Sexual

## Abstract

analizar evidencias sobre la efectividad de intervenciones audiovisuales en el aumento del conocimiento sobre Infecciones Sexualmente Transmisibles en diferentes intervalos de edad.

revisión sistemática, realizada en 21 de febrero de 2023, en la *Cumulative Index to Nursing and Allied Health Literature*, *Cochrane*, Embase, Literatura Latinoamericana y del Caribe en Ciencias de la Salud, Base de Datos en Enfermería, *Índice* Bibliográfico Español en Ciencias de la Salud, MEDLINE, *Scopus* y *Web of Science*. Fueron incluidos estudios experimentales que utilizaron recursos audiovisuales para mejorar el conocimiento, comparados con otras intervenciones o con ninguna. La síntesis de los resultados fue narrativa, con evaluación del riesgo de sesgo y certeza de las evidencias.

la muestra fue de 25 estudios (10 ensayos clínicos aleatorizados y 15 estudios casi-experimentales). El video educativo fue el recurso más utilizado. En la comparación con otra intervención o ninguna, el grupo Intervención alcanzó mejorías significativas en el conocimiento a medio y largo plazo, además de cambios en la percepción y en la predisposición para reducción de comportamientos de riesgo. La certeza de las evidencias fue baja para los estudios aleatorizados y muy baja para los casi-experimentales.

las intervenciones audiovisuales mostraron efectividad en diferentes poblaciones e intervalos de edad, destacándose la necesidad de considerar parámetros específicos en la creación de esos recursos.

## Destacados

**(1)** Eficacia de las intervenciones audiovisuales en la mejoría del conocimiento sobre IST. **(2)** Cambios de actitud y reducción de comportamientos de riesgo después de la intervención. **(3)** Capacidad de usabilidad de las intervenciones para diferentes poblaciones. **(4)** Los recursos audiovisuales son aceptados como herramienta de promoción y prevención de la salud. **(5)** Uso de modelos teóricos para construcción de las intervenciones.

## Introducción

Las Infecciones Sexualmente Transmisibles (IST) han despertado, alrededor del mundo, un gran interés para combatirlas y enfrentarlas. Esfuerzos internacionales han sido realizados a fin de alcanzar tasas inferiores de incidencia de diversas ISTs, como la sífilis, gonorrea, hepatitis y el HIV/SIDA^(1)^. Entre ellos, se destaca la inversión en acciones innovadoras de prevención y concientización, como por ejemplo la oferta de educación en salud para diferentes poblaciones.

Existe evidencia de que se constata un bajo alfabetismo en salud, relacionado a un alto comportamiento de riesgo para las IST en la población general^(2)^, lo que contribuye para una menor percepción de riesgo. Por otro lado, cuando se eleva el nivel de alfabetización en salud, aparece un aumento en la predisposición para la adopción de comportamientos preventivos; por ejemplo, el uso del preservativo^(3)^, lo que reduce los casos de IST.

En el contexto de la educación en salud, es notorio que la modalidad Tecnología Educacional (TE), ha ganado espacio como un recurso para aumentar el conocimiento en salud, con destaque para los recursos audiovisuales. Como el propio nombre sugiere, estos envuelven el uso combinado de imagen y sonido. Algunos ejemplos incluyen videos, presentaciones de *slides*, animaciones, realidad virtual, transmisiones al vivo, holografía, multimedia interactiva, entre otros^(4)^.

Cuando aplicados en el contexto de la educación en salud, ese tipo de intervención ha proporcionado significativos efectos en el aumento del conocimiento y percepción de la salud^(5)^. Esos resultados también son coherentes cuando aplicados, específicamente, en el contexto de las ISTs^(6)^, demostrando que existen evidencias de la contribución de recursos audiovisuales en la oferta de la educación en salud sexual.

Delante de eso, se verifica que el consumo de videos, en el contexto de la salud sobre IST, tuvo un aumento en los últimos años entre adultos, principalmente por medio de las redes sociales, al mismo tiempo en que favoreció la concientización y divulgación del conocimiento de forma efectiva^(7)^. A medida que la tecnología audiovisual se torna cada vez más presente en lo cotidiano, es crucial entender como esas herramientas han sido aplicadas para la educación en salud, así como el aumento de la calidad de las intervenciones y sus efectos.

De ese modo, esta revisión tiene como objetivo analizar evidencias sobre la efectividad de las intervenciones audiovisuales en el aumento del conocimiento sobre ISTs, en diferentes intervalos etarios.

Se espera que sea posible obtener *insights* valiosos para los profesionales de la salud, educadores y formuladores de intervenciones, a fin de contribuir con la construcción de estrategias más eficientes y adaptadas a las necesidades contemporáneas de prevención y promoción de la salud sexual.

## Método

### Diseño del estudio

Esta revisión sistemática de efectividad fue realizada de acuerdo con las etapas metodológicas elaboradas por la JBI^(8)^ y relatada conforme el *Reporting Items for Systematic Reviews and Meta-Analyses* (PRISMA)^(9)^. Este estudio está registrado en el PROSPERO con el número ID CRD42022374619.

### Modificaciones en el protocolo *a priori*

Esta revisión fue realizada con las siguientes modificaciones en el protocolo *a priori*:

- No se utilizaron los instrumentos estandarizados de la JBI para evaluación del riesgo de sesgo, se optó por utilizar las herramientas RoB 2 (*Risk of Bias 2*) y ROBINS-I (*Risk Of Bias In Non-randomized Studies - of Interventions*) de la *Cochrane*. Esa decisión fue tomada con base en el amplio reconocimiento de su evaluación en estudios aleatorizados y no aleatorizados. Como la revisión incluyó ambos, esas herramientas fueron consideradas las más apropiadas para una evaluación precisa y consistente. Además de ser ampliamente utilizadas en la literatura, son complementares a los principios del método JBI y fueron aplicadas de forma rigurosa y transparente, sin comprometer la integridad metodológica de la revisión;

- Se incluyeron, cuando presentes, los resultados secundarios de actitudes y comportamentos, además del resultado primario conocimiento;

- Los datos fueron extraídos por medio de un instrumento creado por los autores, con base en las características de los estudios incluidos;

- Apenas Ensayos Controlados Aleatorizados (ECAs) y los estudios casi-experimentales fueron incluidos. Anteriormente se previó la inclusión de estudios de series temporales y delineamientos observacionales analíticos. Sin embargo, durante el proceso de selección y evaluación de los estudios, se observó que los ECAs y ensayos no aleatorizados atenderían mejor al objetivo del estudio, posibilitando una evaluación más directa y robusta de la efectividad de intervenciones, debido a que hacen comparaciones con grupos pre-establecidos, minimizando sesgos y confusiones. Además, la inclusión de estudios con diseños muy heterogéneos podría comprometer la consistencia de los resultados y la síntesis de las evidencias.

### Pregunta de revisión

La pregunta fue formulada según la estrategia PICO, con: P (Población: jóvenes, adultos y personas mayores); I (Intervención: recursos audiovisuales); C (Comparación: otras intervenciones); y, O (*Outcomes*: conocimiento sobre IST). De esa forma, la siguiente cuestión fue elaborada: ¿Cuál es la efectividad de las intervenciones audiovisuales, cuando comparadas con otros tipos de intervenciones, en el aumento del conocimiento sobre las ISTs en jóvenes, adultos y adultos mayores?

### Criterios de elegibilidad

Se incluyeron ECAs y estudios casi-experimentales. No se realizaron búsquedas directas en la literatura gris. De esa forma, ella fue excluida debido a la variabilidad en la calidad metodológica y a la falta de revisión por pares formales, lo que puede comprometer la confiabilidad de las evidencias.

La población abarcó personas jóvenes, adultos y adultos mayores^(10)^. Es importante destacar que no todos los artículos incluidos optaron por intervalos de edad específicos; pero se enfatiza que no ocurrió cualquier tipo de exclusión con base en cuestiones de edad. Además de eso, no hubo restricción en cuanto al sexo, escolaridad, renta, raza o condición de salud.

En lo que se refiere al tipo de intervención, fueron consideradas apenas aquellas que utilizaron recursos audiovisuales, o sea, estructuradas con el uso de sonido e imágenes asociadas. Eso incluyó videos educativos, programas multimedia, películas tipo documentarios, corto-metrajes y DVDs interactivos. Intervenciones compuestas apenas por llamadas de audio sin video, como en el caso de las fotonovelas o radionovelas, fueron excluidas.

Para la comparación del efecto de la intervención principal, fue considerado cualquier otro tipo de recurso, independientemente de ser o no apoyado en media audiovisual.

El resultado principal de esta revisión fue el nivel de conocimiento sobre ISTs, medido antes y después de la intervención (evaluado por instrumentos estructurados). Los resultados secundarios fueron aquellos relacionados a cambios de comportamiento (acciones físicas o prácticas que contribuyen para la prevención) y a actitudes (posturas mentales o emocionales) frente las ISTs (medidos por escalas o relatos auto-referidos).

Para los estudios no hubo limitación por idioma o año de publicación.

### Fuentes de información y estrategia de búsqueda

Para verificar la adecuación de la pregunta de revisión, así como la existencia de revisiones concluidas o en andamiento sobre la temática, fue realizada una búsqueda inicial en: PROSPERO, *JBI Evidence Synthesis* y MEDLINE (*PubMed*). No habiéndose encontrado revisiones sistemáticas sobre el tema, se procedió con la búsqueda por los términos de indexación principales en el *Medical Subject Headings* (MeSH) y Descriptores en Ciencias de la Salud (DeCS).

Las bases de datos investigadas fueron la *Medical Literature Analysis and Retrieval System on-line* (MEDLINE vía *PubMed*), *Scopus* (vía *Elsevier*), *Web of Science* (vía *Clarivate Analytics*), *Cumulative Index to Nursing and Allied Health Literature* (CINAHL a través de EBSCOhost), Literatura Latino-Americana y del Caribe en Ciencias de la Salud (LILACS vía BVS), *Índice Bibliográfico Español en Ciencias de la Salud* (IBECS a través de BVS), Base de datos de Enfermería (BDENF a través de BVS), Cochrane (a través de *Wiley*) y Embase (a través de *Elsevier*).

La estrategia fue desarrollada inicialmente en la MEDLINE y posteriormente adaptada para cada base de datos, de acuerdo con sus especificaciones y/o descriptores específicos. La estrategia de búsqueda fue estructurada utilizando una combinación de términos adecuados, agrupados por categorías: población (jóvenes, adultos y adultos mayores), intervenciones (audiovisuales) y resultados (ISTs). Fue realizada una búsqueda inicial el 11 de enero y una final (actualización) el 21 de febrero de 2023.

De esta forma, la expresión de búsqueda adoptada fue la siguiente: *# 1”Adolescent”[Mesh] OR Adolescent OR “Young Adult”[Mesh] OR (Young Adult) OR “Adult”[Mesh] OR Adult OR “Aged”[Mesh] OR Aged OR Adolescents OR Adolescence OR Teens OR Teen OR Teenagers OR Teenager OR Youth OR Youths OR (Adolescents, Female) OR (Adolescent, Female) OR (Female Adolescent) OR (Female Adolescents) OR (Adolescents, Male) OR (Adolescent, Male) OR (Male Adolescent) OR (Male Adolescents) OR (Adult, Young) OR (Adults, Young) OR (Young Adults) OR Adults OR Elderly AND #2 “Audiovisual SIDA”[Mesh] OR (Audiovisual SIDA) OR “Video-Audio Media” [Publication Type] OR (Video-Audio Media) OR “Webcast” [Publication Type] OR Webcast OR (Aid, Audiovisual) OR (SIDA, Audiovisual) OR (Audiovisual Aid) OR (Audio-Visual SIDA) OR (Aid, Audio-Visual) OR (SIDA, Audio-Visual) OR (Audio Visual SIDA) OR (Audio-Visual Aid) OR (Visual SIDA) OR (Aid, Visual) OR (SIDA, Visual) OR (Visual Aid) OR (Audiovisual Media) OR (Audio-Visual Media) OR Webcasts OR (Streaming Video) AND #3 “Sexually Transmitted Diseases”[Mesh] OR (Sexually Transmitted Diseases) OR “HIV”[Mesh] OR (HIV) OR “Syphilis”[Mesh] OR Syphilis OR “Hepatitis B”[Mesh] OR (Hepatitis B) OR “Chlamydia”[Mesh] OR Chlamydia OR “Gonorrhea”[Mesh] OR Gonorrhea OR “Trichomonas Infections”[Mesh] OR (Trichomonas Infections) OR “Herpes Genitalis”[Mesh] OR (Herpes Genitalis) OR “Papillomavirus Infections”[Mesh] OR (Papillomavirus Infections) OR (Disease, Sexually Transmitted) OR (Venereal Diseases) OR STDs OR (Sexually Transmitted Infections) OR (Infection, Sexually Transmitted) OR STIs OR STI OR (Human Immunodeficiency Virus) OR (AIDS Virus) OR (Great Pox) OR (Hepatitis B Virus Infection).*

Las búsquedas y el acceso a las bases fueron realizadas por dos revisores, que utilizaron acceso remoto institucional de la Universidad Federal de Piauí (Teresina, Piauí, Brasil) por medio del Portal de Periódicos de la Coordinación de Perfeccionamiento de Personal de Nivel Superior (CAPES). La lista final de referencias de los artículos incluidos fue consultada y los autores correspondientes de los artículos que no estaban disponibles integralmente fueron contactados, cuando fue posible identificar un medio de comunicación.

### Selección de estudios

Los estudios recuperados fueron exportados para el *EndNote* (*Clarivate Analytics*, PA, USA) para excluir duplicaciones. En seguida, los datos fueron transferidos para el *software Rayyan*, donde se realizó la etapa de selección por medio de la lectura de títulos y resúmenes, así como se aplicaron los criterios de elegibilidad.

Dos revisores independientes, acompañados por un tercero, participaron del proceso de clasificación de forma ciega. Los artículos elegibles fueron separados para lectura integral y aplicación nuevamente de los criterios de selección. Aquellos que no atendieron los requisitos preestablecidos fueron excluidos, y los motivos fueron registrados. Los resultados del proceso de selección e inclusión se presentan integralmente por medio del diagrama de flujo disponible en el PRISMA^(9)^, en la sección de resultados.

### Recolección de datos, variables y medidas de efecto

El proceso de recolección de datos fue realizado de manera estructurada, garantizando la integridad y la precisión de las informaciones extraídas de los estudios incluidos en la revisión. Esa etapa, también, fue realizada por dos revisores independientes que utilizaron un formulario de recolección de datos desarrollado para esa finalidad. Después de la extracción inicial, los datos fueron registrados en una planilla organizada para permitir comparaciones y revisiones. Cuando aparecieron dudas en relación a la extracción de informaciones entre los revisores, un tercer revisor fue contactado para esclarecimiento.

El formulario utilizado abordaba las siguientes variables: autores, año y local de publicación, tamaño de la muestra, características de los participantes (edad, sexo, raza, escolaridad y condición de salud, en caso de existir), tipo de estudio, IST para la cual se destinaba la intervención, tipo de recurso audiovisual utilizado en los Grupos de Intervención (GI) y Grupos de Control (GC), contenido de la intervención, tipo de instrumento para evaluación del pretest y postest, tiempo de seguimiento, medidas de resultado, medidas de efecto para datos continuos y/o dicotómicos (*odds ratio*, diferencia media e Intervalo de Confianza - IC), significación estadística y otros indicadores de riesgo de sesgo (randomización, ocultación de asignación, cegamiento e integridad del informe).

Se destaca que los estudios incluidos presentaron las informaciones necesarias para llenar el instrumento de forma cualitativa. En los casos de datos cuantitativos esenciales para evaluación del conocimiento, en algunos estudios esa información apareció de forma generalizada, con enfoque apenas en la significación estadística. Se constató que los autores solicitaron datos ausentes, sin embargo no hubo retorno. Algunos estudios, debido al tiempo de publicación o al propio formato del periódico, no disponían de medios de contacto con el autor correspondiente.

El efecto fue evaluado, para cada resultado cuando disponible, por medio de la diferencia media después de la intervención final, *odds ratio*, significación estadística e IC.

### Evaluación del riesgo de sesgo

La evaluación del riesgo de sesgo fue realizada tanto para los ensayos clínicos aleatorizados como para los estudios casi-experimentales, utilizando las herramientas disponibles en *Cochrane*: *Revised Cochrane Risk-of-Bias tool for Randomized Trials* (RoB 2)^(11)^ y en *Risk Of Bias In Non-randomized Studies - of Interventions* (ROBINS-I)^(12)^, respectivamente. La ROB 2 evalúa cinco dominios, que son: sesgo de asignación, sesgo de desempeño, sesgo de detección, sesgo de relato y sesgo de selección de informes. Por otro lado, la ROBINS-I abarca siete dominios, que incluyen: sesgo de confusión, sesgo de selección de los participantes, sesgo de clasificación de la intervención, sesgo de desviación del protocolo, sesgo de recolección de datos, sesgo de falta de datos y sesgo de relato selectivo.

Ese proceso fue realizado por dos revisores independientes y se presenta detalladamente en la sección de resultados. Independientemente de la calidad metodológica, todos los estudios fueron sometidos al proceso de síntesis y extracción de datos, siendo posteriormente incluidos en esta revisión.

### Síntesis de los resultados

Debido a la variabilidad y heterogeneidad de los artículos incluidos, así como a la ausencia de datos cuantitativos completos, no fue posible realizar un metaanálisis. La mayoría de los estudios relató los resultados de forma cualitativa, sin suministrar suficientes valores numéricos que permitiesen la combinación estadística de los datos.

Además de eso, la falta de estandarización en los resultados (medidos por instrumentos de conocimiento diferentes) y la descripción incompleta de las intervenciones audiovisuales en algunos estudios limitaron la comparación entre los estudios. Así, la síntesis de los datos se presenta de forma narrativa.

De esa forma, para facilitar el análisis comparativo de los resultados, los estudios fueron agrupados de acuerdo con el tipo de estudio. Cada uno fue organizado por el tipo de intervención audiovisual (videos educativos, programas multimedia, DVDs), instrumento de evaluación del conocimiento y los resultados medidos, incluyendo el aumento del conocimiento sobre ISTs y cambios en las actitudes y comportamientos.

Se adoptó el *Grading of Recommendations Assessment, Development and Evaluation Working Group* (GRADE)^(13)^, por medio del *software GRADEpro*^(14)^, para evaluación de la certeza de las evidencias. El análisis fue elaborado para cada resultado de acuerdo con el tipo de estudio.

## Resultados

### Inclusión de los estudios

En este estudio, la búsqueda por evidencias fue realizada por medio de consulta a bases de datos electrónicas y, adicionalmente, por una búsqueda manual en las listas de referencias de los estudios incluidos. Sin embargo, la búsqueda manual no resultó en la identificación de nuevos estudios elegibles para ser incluidos en la revisión. De esa forma, se optó por adaptar el diagrama de flujo PRISMA, excluyendo la etapa de “Identificación de estudios por otros métodos”, una vez que esta no generó adiciones al proceso de selección (Figura 1).

Las búsquedas en las bases de datos resultaron en 2.759 estudios; de esos, 371 fueron removidos por estar duplicados. En seguida, 2.388 artículos fueron seleccionados para lectura de títulos y resúmenes. Mediante la aplicación de los criterios de selección, la muestra final estuvo compuesta por 25 estudios, siendo 10 ECAs y 15 estudios casi-experimentales. El proceso de selección y los motivos de exclusión son presentados detalladamente en la Figura 1.

Durante la lectura integral, cinco estudios no pudieron ser recuperados. De estos, dos fueron retirados de circulación, y tres permanecieron inaccesibles en la íntegra, inclusive después de intentos de contacto con los autores correspondientes.

Además de eso, la búsqueda en la LILACS recuperó una tesis de doctorado que abordaba la misma intervención educativa presentada en uno de los artículos incluidos en esta revisión. Sin embargo, después de una evaluación cuidadosa, se verificó que la tesis no había sido publicada en un periódico revisado por pares. Delante de eso, se optó por excluirla a fin de mantener la consistencia metodológica y los criterios de elegibilidad predefinidos. Detalles adicionales sobre la tesis y su relación con el estudio publicado están descritos en la sección de discusión.

**Figura 1-:** Diagrama de flujo del proceso de selección de los estudios incluidos en la revisión sistemática adaptado del *Preferred Reporting Items for Systematic Review and Meta-Analyses* (PRISMA) (n = 25). Teresina, PI, Brasil, 2024

Fuente: Adaptado^(9)^

### Evaluación del riesgo de sesgo

La evaluación del riesgo de sesgo en los estudios presentó variaciones, indicando diferentes niveles de certeza sobre la validez de los resultados obtenidos. En el caso de los ECAs, se observó que tres estudios presentaron bajo riesgo de sesgo, en cuanto siete presentaron alto riesgo. Entre esos, un estudio demostró alto riesgo de sesgo debido al proceso de randomización (D1), la aleatoriedad no fue garantizada; uno debido a desvíos de las intervenciones pretendidas (D2), ya que los participantes estaban sientes de la intervención recibida; uno debido a la falta de datos de resultados (D3), evidenciado por la significativa pérdida de participantes durante el seguimiento. Además de eso, siete estudios revelaron alto riesgo de sesgo en la mensuración del resultado (D4), ya que los evaluadores de los resultados estaban sientes de la intervención aplicada a los GI y GC (Figura 2).

En lo que se refiere a los estudios casi-experimentales, nueve presentaron riesgo de sesgo moderado, tres presentaron riesgo serio y dos presentaron riesgo de sesgo crítico. Los dominios más susceptibles de sesgo fueron: D3 - riesgo de sesgo en la clasificación de las intervenciones, abarcando 12 artículos, y D6 - riesgo de sesgo en la medición de los resultados, envolviendo 13 artículos. Entre las principales limitaciones de los estudios casi-experimentales incluidos, se destacan la dificultad en enmascarar el conocimiento del resultado, dada la naturaleza de intervención educativa evaluada por pruebas de conocimiento y la ausencia de GC; además de no haber acompañamiento completo de los participantes en los estudios con seguimiento. Adicionalmente, se observó el conocimiento de la intervención recibido y el potencial para el conocimiento de los evaluadores del resultado en relación al GI (Figura 3).

**Figura 2-:** Evaluación del riesgo de sesgo de los ensayos clínicos controlados aleatorizados en cada dominio de la herramienta *Revised Cochrane risk-of-bias tool for randomized trials* (RoB 2) (n = 10). Teresina, PI, Brasil, 2024

Fuente: Imagen creada por el programa Robvis^(15)^

**Figura 3-:** Evaluación del riesgo de sesgo de los estudios casi-experimentales en cada dominio de la herramienta *Risk Of Bias In Non-randomized Studies - of Interventions* (ROBINS-I) (n = 15). Teresina, PI, Brasil, 2024

Fuente: Imagen creada por el programa Robvis^(15)^

### Escenario y año de los estudios incluidos

Los estudios incluidos presentaron variación en el año de publicación, siendo que el estudio más antiguo fue de 1988^(16)^, en cuanto que el más reciente fue de 2022^(6)^. En relación a los locales de realización de las investigaciones, 13 (52%) fueron realizados en los Estados Unidos^(16-28)^, cuatro (16%) en Indonesia^(29-32)^, uno (4%) en cada uno de los siguientes países: Rusia^(33)^, Perú^(34)^, Brasil^(35)^, Canadá^(36)^, Puerto Rico^(37)^, Serbia^(38)^, México^(6)^ y no especificado (estudio *online*)^(39)^.

### Participantes

Los 25 estudios analizados totalizan 6.021 participantes, de los cuales 2.176 pertenecen a los 10 ECAs^(6,16,19,21,23-25,27-28,37)^ y 3.845 a los estudios casi-experimentales^(17-18,20,22,26,29-35,37-39)^.

Entre los participantes, se observaron variaciones significativas. Tres estudios fueron realizados con estudiantes universitarios^(17-18,37)^, en cuanto ocho estudios se enfocaron en adolescentes, con intervalo etario variando de ≤ 13 años a 21 años^(19,25,28,30,32-36)^, siendo uno de ellos también abordó a los cuidadores^(28)^. Cuatro estudios fueron realizados con personas viviendo con alguna IST^(16,20,22,37)^, y un estudio fue desarrollado con hombres con una deficiencia intelectual de leve a moderada^(26)^.

También fueron incluidos participantes adultos atendidos en un departamento de emergencia^(21)^, hombres que hacen sexo con hombres^(23)^, latinos hispanoparlantes^(27)^, usuarios de *Internet*^(39)^, soldados de las fuerzas armadas^(38)^, profesionales de la salud^(31)^ y jóvenes mayores de 18 años^(6)^. Vale resaltar que la mayoría de los estudios abordó exclusivamente el sexo masculino^(16,23-24,26,38)^, otros dos fueron desarrollados con el sexo femenino^(19,29)^. No fue identificado ningún estudio realizado exclusivamente con adultos mayores; la edad más avanzada relatada en el público general fue de 70 años^(20)^.

### Intervenciones/comparadores

Las intervenciones aplicadas serán abordadas de acuerdo con el tipo de estudio. Entre los ECA, la modalidad más adoptada en el GI fue el uso de videos. Cinco de ellos se apoyaron a penas en el uso de videos educativos, enfocando en la disponibilidad de informaciones sobre gonorrea (*It Just Happens Sometimes*)^(16)^, HIV/SIDA (*Do you know about rapid HIV testing?)*^(21)^; video educacional en español sobre HIV/SIDA)^(27)^, sífilis (*Syphilis and Men*)^(23-24)^ y HPV (¡*7* cosas que debes saber sobre el *VPH!*)^(6)^. Un video fue interactivo sobre IST de forma general^(19)^, proporcionando no solo informaciones, pero también elementos que permitía a los participantes realizar acciones.

Además de eso, un video se concentró en el aspecto educacional y en consejos pretest del HIV^(25)^. Otra intervención combinó el uso de panfleto educativo y video para tratar sobre el HPV y la vacunación^(36)^. Por último, hubo una intervención que utilizó un DVD en conjunto con un libro de actividades^(28)^, proporcionando un abordaje más extenso. Este cubrió diversos módulos para promover la comunicación, prevención y habilidades interpersonales relacionadas al HIV/SIDA (*Work It Out Together*).

El tiempo de duración de las intervenciones también varió, con la mayoría concentrándose en un intervalo inferior a 10 minutos. El menor tiempo registrado fue de tres minutos^(6)^, en cuanto el mayor alcanzó una hora^(19)^.

Entre los comparadores, GC, se destaca que dos no recibieron ninguna intervención^(6,16)^, uno recibió el cuidado estándar de atención sin ser sometido a una intervención^(23)^, dos recibieron materiales de lectura^(19,36)^, tres fueron sometidos a sesiones de consejos presenciales^(21,25,27)^, uno recibió un *palm card* (cartón como parte de la campaña de marketing social en respuesta a la epidemia de sífilis)^(24)^, y uno fue expuesto a un DVD de promoción de la salud general que no poseía un enfoque específico en la reducción de riesgos relacionados al comportamiento sexual o uso de sustancias^(28)^. La síntesis de los ECA por tipo de intervención se presenta en la Figura 4.

En los estudios casi-experimentales, la modalidad de video también fue la más utilizada, abarcando videos informativos, educacionales y de tipo documentario. Tres intervenciones tuvieron como base el uso de programas educacionales multimedia^(22,37)^, siendo uno de ellos interactivo y accesible para personas con deficiencia intelectual^(26)^. Una intervención adoptó el formato de película, del tipo corto metraje^(38)^, en cuanto otra trató de un programa gubernamental que utilizó un audiovisual aplicado por medio de sesiones de discusión sobre la temática del HIV/SIDA^(30)^.

Hubo una gran variación en relación al tiempo de duración de las intervenciones, siendo que algunos estudios no suministraron una descripción detallada. El menor tiempo registrado fue de 15 minutos^(39)^, en cuanto el mayor relató una duración de dos horas^(33)^. En relación a los GC, nueve estudios no incluyeron un GC, cuatro no aplicaron ninguna intervención en el grupo de comparación y apenas tres compararon la intervención principal con otra (Figura 5).

Pocos fueron los estudios que describieron el medio de transmisión por el cual las intervenciones quedaron disponibles para los participantes, como la intervención estaba de hecho estructurada, así como si eran accesibles de forma física o electrónica y si contenían elementos de accesibilidad para poblaciones con alguna deficiencia. Más informaciones sobre las intervenciones están disponibles en las Figura 4 y 5.

Los referenciales teóricos utilizados en la intervención relatados en los estudios incluyeron: Teoría del aprendizaje social de Bandura^(26)^, *Health Belief Model* (HBM)^(16,22,37)^, *Social Cognitive Theory* (SCT)^(22,37)^, *Health media and marketing*^(24)^, modelo teórico de modelos mentales simbólicos y ensayo cognitivo^(19)^, metodología constructivista basada en problemas y en el pensamiento crítico de Albion y Gibson^(34)^, Teoría de la promoción de la salud, Teoría del comportamiento planificado y Teoría de la creencia de salud^(36)^ y Modelo de información, motivación y habilidades comportamentales de Fisher y Fisher^(6)^.

## Resultado

Los estudios abordados presentan una diversidad de ISTs, 14 estudios se enfocaron en el HIV/SIDA^(17-18,20,22,26,29-35,37-39)^, en cuanto dos estudios abordaron el HIV y las ISTs de forma asociada^(28,34)^. Dos estudios trataron del virus de la hepatitis C^(22,37)^, un estudio abordó las ISTs de forma general^(19)^, un estudio abordó la gonorrea^(16)^, dos estudios trataron de la sífilis^(23-24)^, y dos estudios trataron del HPV^(6,36)^. El HIV ha sido la IST más explorada en el contexto de intervenciones educativas audiovisuales (Figura 4 y 5).

En relación al resultado primario, todos los estudios evaluaron el conocimiento por medio de instrumentos aplicados antes y después de la intervención, hubo una mejora significativa (p<0,05) de esas variables en el postest para los grupos de tratamiento; entre tanto, dos estudios, entre los aleatorizados controlados, no presentaron cambios significativos después de la aplicación de la intervención^(6,27)^. Entre los casi-experimentales, a pesar de cambios estadísticamente significativos en las puntuaciones de conocimiento, dos estudios evaluaron que la magnitud de esos cambios fue modesta^(17-18)^ (Figura 4 y 5).

No había descripción en los estudios sobre como el formato en que las intervenciones fueron creadas impactó en la mejora del conocimiento. Apenas un estudio relató que un abordaje de presentación más científico mostró resultados mejores en la evaluación del conocimiento que otro más informal^(35)^.

Apenas 11 estudios trataron otros resultados además del conocimiento. La mayoría envolvió la intervención en cambios de actitudes, se destacan mejorías en la adhesión al test para el HIV^(25)^, vacunación contra el HPV^(36)^, reducción de comportamientos sexuales de riesgo^(18-19,28)^ y uso de sustancias^(28)^. Hubo mejora en la autoeficacia percibida en relación a la adhesión al tratamiento^(20)^, mejoría en las habilidades relacionadas al HIV/SIDA y uso de preservativos^(26)^, aumento del comportamiento de protección contra el HIV^(29)^ y cambios de actitud frente al HIV/SIDA^(31-32)^. No fueron encontradas alteraciones en la predisposición para notificar contactos sexuales sobre el diagnóstico de IST^(16)^. Apenas un estudio trató cambios de comportamiento, enfocado en la reducción del compartir jeringas y equipamientos de preparación de drogas^(37)^ (Figura 4 y 5).

**Figura 4-:** Síntesis de los ensayos clínicos controlados aleatorizados incluidos (n = 10). Teresina, PI, Brasil, 2024

**Autores, año y loc** al	**Muestra/tiempo de seguimiento**	GI*/tipo de tecnología educativa	**GC** ^†^ **/tipo de tecnología**	**Instrumento de evaluación del conocimiento**	**Principales resultados**
Solomon, DeJong, 1988. EUA ^( 16 )^	n=175/14 días	**Video:** “ *It Just Happens Sometimes* ” (10 min; n=83) Tipo/IST ^‡^ : Video educativo/Gonorrea Contenido: Personajes discutiendo síntomas, tratamiento y prevención, con énfasis en asesoramiento para el diagnóstico. Público objetivo: Hombres negros con gonorrea.	Grupo sin video educativo (n=92).	Test verdadero y falso de 18 ítems.	**Conocimiento:** Media de aciertos GI* 16,6 (de ^§^ = 1,7), GC ^†^ 13,3 (de ^§^ = 2,2; 1(239) =13,4, *p* ^||^ <0,0001). **Actitud:** Disposición para notificar contactos sexuales ( *p* ^||^ >0,05) entre GI* y GC ^†^ .
Downs, et al. 2004. EUA ^( 19 )^	n=300/6 meses	**Video:** (1 h; n=no explícito) Tipo/IST ^‡^ : Video interactivo/IST ^‡^ Contenido: Comportamientos de negociación, eficacia del uso de preservativos, anatomía reproductiva femenina y respuestas fisiológicas a infecciones. Público objetivo: Adolescentes del sexo femenino.	Materiales de lectura (n=no explícito).	Test verdadero y falso de 40 ítems.	**Conocimiento:** Diferencia media entre GI* y GC ^†^ , 5,3 puntos y 5,1 puntos, respectivamente. Mejorías significativas en el conocimiento a lo largo del tiempo (general: F ^¶^ (1,214= 69,09, *p* ^||^ <0,001; específico: F ^¶^ (1,214)= 58,62, *p* ^||^ <0,001).
Merchant, et al. 2009. EUA ^( 21 )^	n=574/no tuvo seguimiento	**Video:** “ Do you know about rapid HIV** testing ?” (9,5 min; n=270) Tipo/IST ^‡^ : Video educativo/HIV** Contenido: Definición, transmisión, prevención y test. Público objetivo: Adultos que frecuentaban una urgencia y emergencia.	Sesión presencial con consejero del test de HIV** (n=304).	Cuestionario “Rapid HIV** Pretest Information Comprehension ” de 26 preguntas.	**Conocimiento:** Diferencia media GI* vs. GC ^†^ **=** 0,68 (IC ^††^ 95%; 0,18-1,26; *p* ^||^ <0,05). Las puntuaciones medias diferían por menos de una pregunta respondida correctamente.
Sánchez, et al. 2010. EUA ^( 23 )^	n=206/no tuvo seguimiento	**Video:** “ *Syphilis and Men* ” (5 min; n=100) Tipo/IST ^‡^ : Video educativo/Sífilis Contenido: Transmisión, síntomas, tratamiento, conexión entre sífilis-HIV** y reducción de riesgo. Público objetivo: Hombres que frecuentaban una urgencia y emergencia.	Cuidado-estándar sin video sobre sífilis (n=106).	Cuestionario: 10 cuestiones de múltiple elección.	**Conocimiento:** GI* puntaje medio 24,8% más alto que GC ^†^ en el test post intervención ( *p* ^||^ < 0,001).
Sánchez, Guilliames, et al. 2010. EUA ^( 24 )^	n=168/ no tuvo seguimiento	**Video:** “ *Syphilis and Men* ” (5 min; n=85) Tipo/IST ^‡^ : Video educativo/Sífilis Contenido: Transmisión, síntomas, tratamiento, conexión entre sífilis-HIV** y reducción de riesgo. Público objetivo: Hombres negros que hacen sexo con hombres.	*“Syphilis is Back” Palm Card* (n=83).	Cuestionario desarrollado por los investigadores (10 cuestiones de opción múltiple).	**Conocimiento:** GI* 8,9 a 9,8 preguntas correctas, GC ^†^ 6,9 a 7,9 preguntas correctas. Aumento de 19,5% para 20,9% en la puntuación del postest para GI* ( *p* ^||^ <0,05).
Calderon, et al. 2011. EUA ^( 25 )^	n=200/ no tuvo seguimiento	**Video:** (4 min, n=100) Tipo/IST ^‡^ : Video educativo/HIV** Contenido: Abarca siete elementos esenciales para una sesión de asesoramiento pretest para HIV**. Público objetivo: Adolescentes	Sesión presencial con consejero de test del HIV** (n=100).	Cuestionario de 10 preguntas creado por Carey y Schroder, y cuestiones del Departamento de Salud del Estado de Nova York.	**Conocimiento:** Diferencia media GI* vs. GC ^†^ = 12,2% ( *p* ^||^ <0,01); GI* 78,5% correctas y GC ^†^ 66,3% correctas. **Actitud:** Aceptar el test para el HIV**: GI*, OR ^‡‡^ = 3,6x (IC ^††^ 95%:1,8–7,2; *p* ^||^ <0,001) comparado con GC ^†^ .
Krawczyk, et al. 2012. Canadá ^( 36 )^	n=200/ no tuvo seguimiento	**Video** (5 min, n=74) **+ Panfleto educacional** (n=61) Tipo/IST ^‡^ : Video + Panfleto/HPV ^§§^ Contenido: El video presenta un médico discutiendo la incidencia, transmisión, consecuencias del virus y eficacia de la vacuna. el panfleto contiene informaciones sobre HPV ^§§^ y vacuna. Público objetivo: Estudiantes universitarios	Apenas panfleto sobre prevención del cáncer de forma general (n=65).	Test de 22 ítems (evaluación: verdadero, falso y no sé).	**Conocimiento:** Media de aciertos GI* (panfleto)= 17,46; GI*(video)= 16,70; GC ^†^ = 12,06 ( *p* ^||^ <0,001). **Actitud:** Aumento en la intención de vacunación para GI* (panfleto) y GI* (video) en comparación con GC ^†^ ( *p* ^||^ < 0,001).
Merchant, et al. 2014. EUA ^( 27 )^	n=150/ no tuvo seguimiento	**Video:** (9,5 min, n=75) Tipo/IST ^‡^ : Video educativo/HIV** Contenido: Definiciones, naturaleza, transmisión, prevención, métodos de test e interpretación de resultados de HIV**. Público objetivo: Latinos hispanohablantes	Información oral del consejero de test de HIV** (n=75).	Test de 25 ítems (Evaluación: si, no, no sé).	**Conocimiento:** Diferencia media GI* vs. GC ^†^ = GI* 20,4 (IC ^††^ 95%: 19,5-21,3); GC ^†^ 20,6 (IC ^††^ 95%: 19,7-21,5), indicando semejanza (IC ^††^ 95%:-1,4-1,1).
Hadley, et al. 2016. EUA ^( 28 )^	n=170/3 meses	**DVD + libro** : “ *Work It Out Together* ” (20 min/módulo; n=83) Tipo/IST ^‡^ : DVD interactivo + libro de actividades/HIV** Contenido: Seis módulos abordando comunicación entre padres y adolescentes, prevención, uso de preservativos, negociación de sexo seguro, habilidades y toma de decisiones. Público objetivo: Adolescentes	DVD de promoción de la salud general (n=87).	Test de conocimiento con 10 ítems (verdadero, falso, incierto).	**Conocimiento:** Media de aciertos GI*= 6,71; GC ^†^ = 6,45. Los padres obtuvieron más puntos (t ^||||^ (158)=2,00, *p* ^||^ <0,05). **Actitud:** GI* (padres) mayor autoeficacia para comprometerse en comportamientos de prevención (t ^||||^ (158)= 2,28, *p* ^||^ <0,05). **Actitud:** GI*(adolescentes) mayor autoeficacia para usar preservativos (t ^||||^ (64)= 2,06, *p* ^||^ <0,05).
Ortíz, et al. 2022. México ^( 6 )^	n=33/7 días	**Video:** “ *7 cosas que debes saber sobre el VPH* ^§§^ *!* ” (3 min; n=16) Tipo/IST ^‡^ : Video educativo/HPV ^§§^ Contenido: Definición, formas de transmisión, síntomas, diagnóstico, tratamiento y prevención. Público objetivo: Jóvenes mayores de 18 años	Grupo sin video educativo (n=17).	Test de 38 ítems (Verdadero, falso y no sé).	**Conocimiento:** GI* superior al GC ^†^ ( *p* ^||^ =0,020 y *p* ^||^ =0,024, respectivamente). Media de aciertos GI*= 30,75 (de ^§^ = 2,32); GC ^†^ 28,18 (de ^§^ = 3,55).

*GI = Grupo Intervención; ^†^GC = Grupo Control; ^‡^IST = Infecciones Sexualmente Transmisibles; ^§^de = Desviación estándar; ^||^*p* = Significación Estadística; ^¶^F = Análisis de Variancia; **HIV = Virus de la Inmunodeficiencia Humana; ^††^IC = Intervalo de Confianza; ^‡‡^OR = *Odds Ratio*; ^§§^HPV = Papiloma virus Humano; ^||||^t = Estadística t (t de Student)

**Figura 5-:** Síntesis de los estudios casi-experimentales incluidos (n = 15). Teresina, PI, Brasil, 2024

**Autores, año y loca** l	**Muestra/tiempo de seguimiento**	GI*/tipo de tecnología educativa	**GC** ^†^ **/tipo de tecnología**	**Instrumento de evaluación del conocimiento**	**Principales resultados**
Rhodes, Wolitski. 1989. EUA ^( 17 )^	n=441/6 semanas	**Videos:** (16-20 min; n=no explícito) Tipo/IST ^‡^ : 4 Videos informativos/SIDA Contenido: (1) *SIDA: Acquired Immune Deficiency Syndrome,* 1986, 18 min; (2) SIDA: *What Everyone Needs to Know* , 1986, 18 min; (3) *Beyond Fear: The Virus,* 1986, 20 min; (4) *Sex, Drugs, and SIDA,* 1986, 16 min. Público objetivo: Estudiantes universitarios.	No observaron ninguno de los videos (n= no explícito).	Test verdadero y falso de 23 ítems.	**Conocimiento:** Aumento en el conocimiento en los cuatro videos ( *p* ^§^ <0,001), superior al GC ^†^ en el postest tardío ( *p* ^§^ <0,01).
Lipson, Brown. 1991. EUA ^( 18 )^	n=144/1 mes	**Video:** (19-40 min; n=90) Tipo/IST ^‡^ : 3 Videos Informativos/SIDA Contenido: (1) *Sex, drugs, and SIDA* , 19 min; (2) *SIDA: beyond fear* , 40 min; (3) *SIDA: what you need to know* , 40 min Público objetivo: Estudiantes universitarios.	Fita de video “ *Where Did I Come From* ?”, aborda la educación sexual, pero no el HIV ^||^ /SIDA (n=54).	Cuestionario de 55 ítems (La respuesta más correcta recibió nota 4, y la menos correcta nota 0).	**Conocimiento:** Diferencia media GI* vs. GC ^†^ = 15,93 puntos ( *p* ^§^ <0,01). **Actitud:** GI* mejorías en las actitudes en relación a los comportamientos preventivos, diferencia media de 1,33 puntos en relación GC ^†^ ( *p* ^§^ <0,01).
Torabi, et al. 2000. Rusia ^( 33 )^	n=1.124/2 semanas	**Video:** (2 h; n=731) Tipo/IST ^‡^ : Video informativo/HIV ^||^ Contenido: Prevención, utilizó técnicas de conferencias, ilustración y preguntas y respuestas. Público objetivo: Adolescentes.	Programa académico usual, omite la educación sobre HIV ^||^ /SIDA (n=393).	Test de 51 preguntas (“si”, “no” y “no tengo seguridad”).	**Conocimiento:** Puntuaciones del GI* en comparación con GC ^†^ fueron superiores (F ^¶^ =58,73, *p* ^§^ <0,01).
Velásquez. 2005. Perú ^( 34 )^	n=454/no tuvo acompañamiento	**CD:** “ *Planeta Riesgo Cero”* (duración no descrita; n=454) Tipo/IST ^‡^ : CD educativo/IST ^‡^ y HIV ^||^ Contenido: Historias cotidianas de adolescentes, juego sobre toma de decisiones, informaciones generales, riesgos, mitos y creencias. Público objetivo: Adolescentes.	No tuvo GC.	Cuestionario de 101 preguntas dividido en cuatro secciones.	**Conocimiento:** Aumento de 3x más en el conocimiento sobre el HIV ^||^ y 6x más en el de SIDA ( *p* ^§^ <0,001). Probabilidad aumentada de conocer gonorrea (OR**=5,4), sífilis (OR**=4,8), clamidia (OR**=13,2), herpes genital (OR**=3,7) y chancroide (OR**=2,3, *p* ^§^ <0,001).
Brock, Smith. 2007. EUA ^( 20 )^	n=51/4 a 6 semanas	**Video:** (25 min; n=51) Tipo/IST ^‡^ : Video educacional/HIV ^||^ Contenido: Exhibido en un *Personal Digital Assistant* (PDA), que contenía informaciones para adhesión a medicación antirretroviral. Público objetivo: Pacientes de ambulatorio de enfermedades infecciosas.	No tuvo GC.	Instrumento con nueve preguntas.	**Conocimiento:** Mejorías en el conocimiento del HIV ^||^ y del tratamiento ( *p* ^§^ <0,005). **Actitud:** Aumento en la autoeficacia para adhesión al tratamiento ( *p* ^§^ <0,005).
Camargo, Barbará, Bertoldo. 2008. Brasil ^( 35 )^	n=141/1 semana	**Videos:** (duración no descrita) Tipo/IST ^‡^ : Videos documentarios/HIV ^||^ Contenido: Video 1, abordaje científico, desarrollo del SIDA, prevención y detalles de la enfermedad (n=46). Video 2, informaciones básicas y declaraciones de adolescentes viviendo con el virus (n=39). Público objetivo: Adolescentes.	No observaron ninguno de los videos (n=56).	Test de conocimiento científico sobre el SIDA.	**Conocimiento:** GI*1 presentó mejor conocimiento en relación al GI*2 y al GC ^†^ (F ^¶^ =8,15; *p* ^§^ <0,001).
Mayor. 2010. EUA ^( 22 )^	n=110/8 semanas	**Programa multimedia:** (25-35 min/sesión; n=110) Tipo/IST ^‡^ : Programa educacional/ HCV ^††^ Contenido: Desarrollado en *PowerPoint* , aborda la susceptibilidad, medidas de protección y relación con el HIV ^||^ . Público objetivo: Pacientes viviendo con HIV ^||^ .	No tuvo GC.	Cuestionario autoaplicable con escala Likert.	**Conocimiento: La** comprensión de que la hepatitis C es una condición viral aumentó de 73,1% para 85,2% ( *p* ^§^ =0,03). Comprensión de que el HCV ^††^ es encontrado predominantemente en la sangre aumentó de 92% para 98,2% ( *p* ^§^ =0,01).
Mayor. 2013. Puerto Rico ^( 37 )^	n=88/6 meses	**Programa multimedia:** (duración no descrita; n=88) Tipo/IST ^‡^ : Programa educacional /HCV ^††^ Contenido: Desarrollado en *PowerPoint* , aborda la susceptibilidad, medidas de protección y relación con el HIV ^||^ . Público objetivo: Usuarios de drogas con HIV ^||^ .	No tuvo GC.	Cuestionario autoaplicable con escala Likert.	**Conocimiento:** Aumento en la concientización sobre manifestaciones clínicas, comportamientos de riesgo, prácticas de prevención y sinergia de coinfección HIV ^||^ /HCV ^††^ ( *p* ^§^ <0,05). **Comportamiento:** Reducción de compartir jeringas y equipamientos de preparación de drogas de 39,8% para 13,6% ( *p* ^§^ <0,001).
Wells, Clark, Sarno. 2014. EUA ^( 26 )^	n=49/no tuvo seguimiento	**Programa multimedia:** (1,5 h, n=49) Tipo/IST ^‡^ : Programa interactivo/HIV ^||^ Contenido: Basado en computador, enfocado en la prevención. Público objetivo: Hombres con deficiencia intelectual leve a moderada.	No tuvo GC.	Cuestionario con 44 preguntas.	**Conocimiento:** Aumento todas las áreas evaluadas, con un tamaño medio del efecto de 0,67 (efecto grande).
Jadranin. 2015. Serbia ^( 38 )^	n=102/ no tuvo seguimiento	**Filme:** *“HIV/SIDA prevention and control in the Serbian Armed Forces”* (17 min, n=102) Tipo/IST ^‡^ : Película educativa/HIV ^||^ Contenido: Prevención, transmisión, progresión de la enfermedad y test. Público objetivo: Soldados de las Fuerzas Armadas.	No tuvo GC.	Cuestionario de 23 preguntas de múltiple elección.	**Conocimiento:** Diferencia media antes y después = 1,91 puntos, con aumento en la puntuación después de la intervención ( *p* ^§^ <0,001).
Shao, 2016. Estudio *online* ^( 39 )^	n=817/6 semanas	**Video:** (15 min, n=817) Tipo/IST ^‡^ : Video informativo/HIV ^||^ Contenido: Utiliza animación para informar sobre infección aguda y métodos de test. Público objetivo: Usuarios de la *Internet* .	No tuvo GC.	Cuestionario de 25 preguntas + una única pregunta en una escala de 4 puntos.	**Conocimiento:** Diferencia media antes y después del video= hablantes de inglés (Post:19,6 vs. Pre:16,4; Δ ^‡‡^ =3,2; IC ^§§^ 95%: 2,8-3,5) y hablantes de español (Post:20,7 vs. Pre:17,3; Δ ^‡‡^ =3,4; IC ^§§^ 95%: 3,0-3,8), con los hablantes de inglés presentando menor conocimiento previo ( *p* ^§^ <0,05).
Dewi, Amir, Nauli. 2019. Indonesia ^( 29 )^	n=144/no tuvo seguimiento	**Video + folletos:** (n=72) Tipo/IST ^‡^ : Video educativo + folleto/HIV ^||^ Contenido: Informaciones para aumentar la comprensión y promover comportamientos de prevención. Público objetivo: Mujeres casadas.	No recibió ninguna de las intervenciones (n=72).	Cuestionario comprobado en cuanto a la validez y confiabilidad.	**Conocimiento:** Diferencia media entre GI* vs. GC ^†^ = 3,1; GI*(12,86) y GC ^†^ (9,69) (IC ^§§^ 95%:2,60- 3,73, *p* ^§^ = 0,000). **Actitud:** Diferencia media de 8,4 para actitud de prevención entre GI* y GC ^†^ (IC ^§§^ 95%: 0,879-6,66, *p* ^§^ =0,000).
Nuramalia, et al. 2020. Indonesia ^( 30 )^	n=96/4 semanas	**Media audiovisual ABAT:** *“I’m Proud I Know”* (120 min/sesión; n=48) Tipo/IST ^‡^ : Media educativa/HIV ^||^ Contenido: Salud reproductiva, drogas, estilo de vida, HIV ^||^ y SIDA. Público objetivo: Adolescentes.	Una única sesión por semana (duración de tres semanas), abordando los mismos temas (n=48).	Cuestionario con preguntas de conocimiento y prácticas de prevención.	**Conocimiento:** Aumento en la categoría “Bueno” para 91,6%, en cuanto la categoría “Suficiente” fue de 8,3%. Hubo diferencias en el conocimiento entre GI* y GC ^†^ ( *p* ^§^ < 0,05).
Prihanti. 2020. Indonesia ^( 31 )^	n=41/no tuvo seguimiento	**Video:** (duración no descrita; n=41) Tipo/IST ^‡^ : Video *online* /HIV ^||^ Contenido: Contiene informaciones sobre la imagen general del HIV, factores de riesgo, transmisión y la importancia del test diagnóstico. Público objetivo: Profesionales de salud.	No tuvo GC.	*E-Questionnaire* vía *Google Form* con 28 preguntas.	**Conocimiento:** 97,6% de los participantes demostraron buen conocimiento y apenas 2,4% mantuvieron conocimiento insuficiente ( *p* ^§^ =0,00). **Actitud:** 85,4% de los participantes demostraron actitudes positivas y 14,6% mantuvieron actitudes negativas ( *p* ^§^ =0,00).
Ambarwati. 2021. Indonesia ^( 32 )^	n=43/no tuvo seguimiento	**Video +** slides **:** (n=43) Tipo/IST ^‡^ : Video educativo + *slides* /HIV ^||^ Contenido: Desarrollados para mejorar el conocimiento y actitudes en relación al HIV ^||^ /SIDA. Público objetivo: Adolescentes.	No tuvo GC.	Cuestionario con preguntas no detalladas.	**Conocimiento:** Aumento en el conocimiento (Z ^||||^ =-3,819; *p* ^§^ =0,000). **Actitud:** Aumento en la actitud después de la intervención (Z ^||||^ =-3,873; *p* ^§^ =0,00).

*GI = Grupo Intervención; ^†^GC = Grupo Control; ^‡^IST = Infecciones Sexualmente Transmisibles; ^§^*p* = Significación Estadística; ^||^HIV = Virus de la Inmunodeficiencia Humana; ^¶^F = Análisis de Variancia; **OR = *Odds Ratio*; ^††^HCV = Virus de la Hepatitis C; ^‡‡^Δ = Estadística Delta; ^§§^IC = Intervalo de Confianza; ^||||^Z = Estadística Z

La evaluación de la certeza de las evidencias de acuerdo con los resultados seleccionados se presenta en la figura 6. La calidad de las evidencias fue clasificada como baja para ECAs y muy baja para estudios casi-experimentales, debido a factores como riesgo de sesgo grave o extremadamente serio, imprecisión (IC amplios o falta de datos cuantitativos) e inconsistencia no grave. La mayoría de los estudios presentó limitaciones metodológicas, como alto riesgo de sesgo e imprecisión, lo que impactó la confiabilidad de los resultados.

**Figura 6-:** Evaluación de la certeza de las evidencias, según el *Grading of Recommendations Assessment, Development and Evaluation* (GRADE). Teresina, PI, Brasil, 2024

**Certeza de la evidencia**							**Número de pacientes**		**Efecto** ^††^		**Certeza**
**Nº de los estudios**	**Diseño del estudio**	**Riesgo de sesgo**	**Inconsistencia**	**Evidencia indirecta**	**Imprecisión**	**Otras consideraciones**	**Recursos audiovisuales**	**Ninguno u otro**	Relativo (95% IC*)	Absoluto (95% IC*)	
**Aumento del conocimiento sobre IST**											
10	ECA ^†^	grave ^‡^	no grave	no grave	grave ^§^	ninguno	886/1815 (48,8%)	929/1815 (51,2%)	no estimable	no estimable	⨁⨁◯◯ Baja ^‡§^
15	Estudios casi experimentales	sumamente serio ^||^	no grave	no grave	grave ^§^	ninguno	2781/3404 (81,7%)	623/3404 (18,3%)	no estimable	no estimable	⨁◯◯◯ Muy baja ^§||^
**Cambio de actitud**											
4	ECA ^†^	grave ^¶^	no grave	no grave	grave ^§^	ninguno	327/671 (48,7%)	344/671 (51,3%)	no estimable	no estimable	⨁⨁◯◯ Baja ^§¶^
5	Estudios casi experimentales	sumamente serio**	no grave	no grave	grave ^§^	ninguno	297/423 (70,2%)	126/423 (29,8%)	no estimable	no estimable	⨁◯◯◯ Muy baja ^§^ **
**Cambio de comportamiento**											
1	Estudios casi experimentales	sumamente serio**	no grave	no grave	grave ^§^	ninguno	88/88 (100,0%)	0/0	no estimable	no estimable	⨁◯◯◯ Muy baja ^§^ **

*IC = Intervalo de Confianza; ^†^ECA = Ensayo Clínico Randomizado; ^‡^Siete estudios presentaron riesgo alto de sesgo de acuerdo con la herramienta ROB-2; ^§^Algunos resultados presentaron intervalos de confianza amplios, indicando imprecisión. Para otros resultados, existe carencia de datos cuantitativos, lo que impide la evaluación completa de la precisión; ^||^Dos estudios presentaron riesgo crítico de sesgo de acuerdo con la herramienta ROBINS-I; ^¶^Tres estudios presentan riesgo alto de sesgo de acuerdo con la herramienta ROB-2; **Un estudio presenta riesgo crítico de sesgo de acuerdo con la herramienta ROBINS-I, ^††^No estimable debido a la variabilidad de los estudios que imposibilitó el metaanálisis

## Discusión

El objetivo de esta revisión sistemática fue analizar las evidencias disponibles sobre la efectividad de intervenciones audiovisuales en el aumento del conocimiento sobre IST en jóvenes, adultos y adultos mayores. Con base en los resultados, fue posible evidenciar que esos recursos contribuyen para una homogeneidad del conocimiento factual, principalmente entre adolescentes y jóvenes-adultos. Esas poblaciones son consideradas de riesgo para las IST, ya que en el intervalo etario de 13 a 34 años se concentra más de la mitad (56%) de los nuevos casos del HIV^(40)^. Luego, las intervenciones han sido direccionadas a la parcela de la población que más necesita de acceso a informaciones sobre la temática.

La mayoría de los estudios incluidos apuntan que las intervenciones educacionales audiovisuales han sido ampliamente exploradas como herramientas eficaces en la promoción del conocimiento sobre IST, especialmente el HIV/SIDA. Además de eso, existe un impacto de estas tecnologías en el cambio de actitud y, en algunos casos, reducción de comportamientos de riesgo relacionados a esas infecciones.

Se destaca que las tecnologías audiovisuales, por ser consideradas parte de las Tecnologías de la Información y Comunicación (TIC), también se clasifican como “tecnología leve-dura”. O sea, ellas se presentan por medio de *hardwares* y dispositivos y necesitan de otros recursos, como cámaras, micrófonos, alto-parlantes, monitores, además de *software* relacionado a la producción y reproducción de contenido audiovisual^(41)^.

Ese tipo de tecnología, “leve-dura”, tiene potencial para despertar el aprendizaje y el cambio de comportamientos en salud, una vez que posibilitan una abordaje capaz de estimular los sentidos y mantener los interesados más firmemente atentos al contenido que ellas se proponen a presentar^(42)^. Se puede inferir que intervenciones apoyadas en ese tipo de clasificación pueden tener efectos satisfactorios en el aumento del conocimiento en salud y en la adopción de prácticas y comportamientos más seguros.

Como descrito, hubo un consenso entre las publicaciones a respecto del impacto del audiovisual en el conocimiento. De forma general y específica, es posible percibir que las informaciones repasadas fueron bien absorbidas por los participantes en el postest^(19)^. Sumado a eso, también se destaca la influencia de videos educacionales en la mejora significativa del conocimiento sobre HIV/SIDA, en comunidades norteamericanas en la cuales el idioma español es dominante^(27)^.

Sin embargo, a pesar de la gran cantidad de resultados mostrando mejorías estadísticamente significativas en el conocimiento, es necesario considerar también que la fuerza de esos cambios puede ser modesta^(17)^. Así, se debe considerar incluida la relevancia clínica, para más allá de la significación estadística. En otras palabras, existe la necesidad de observar esa mejora del conocimiento juntamente con la predisposición de los participantes en reducir comportamientos de riesgo y adoptar medidas de prevención en relación a las IST.

La diversidad de contextos en los cuales los estudios fueron realizados, incluyendo diferentes poblaciones (directa e indirectamente introducidas en el contexto de las IST), intervalos de edad y niveles de alfabetización en salud, se destaca la flexibilidad y la adaptabilidad de las intervenciones con videos educacionales en la promoción de la salud. Sin embargo, la generalización de los resultados para la población en general puede ser limitada, especialmente cuando la población que es objetivo, está inserida en el contexto escolar y posee niveles de alfabetización considerados satisfactorios para la edad que presenta^(35)^.

Pocos fueron los estudios que observaron si el nivel de escolaridad de los participantes puede interferir en el proceso de aprendizaje. Un abordaje de video dinámico, como por ejemplo, el que cuenta una historia basada en personajes sobre la temática de la gonorrea, en el contexto de negociar con las alianzas a respecto de la notificación y exámenes para IST, llevó a todos los pacientes del grupo de tratamiento a un alto nivel de conocimiento^(16)^, independientemente del nivel educacional^(39)^.

Sin embargo, un video, también como una propuesta interactiva, reconoció que los participantes con menor escolaridad pueden necesitar de abordajes adicionales^(23)^. Además de eso, existe una necesidad de considerar barreras culturales y lingüísticas en intervenciones educacionales^(6,24)^. De esa forma, es necesario considerar la alfabetización en salud de la población a la cual se destina la intervención, ya que diferentes poblaciones, dentro de un mismo contexto, pueden presentar niveles diferentes de comprensión de informaciones sobre la salud^(43)^.

En términos de metodología de construcción de la intervención, algunos estudios destacaron el uso de referenciales teóricos para dar base a la estructura de la misma y a su finalidad, desde teorías orientadas para el aprendizaje^(26)^, hasta teorías que predisponen a cambios de comportamiento de forma planificada^(36)^. Se verifica que el uso de una comunicación persuasiva, proveniente de un modelo teórico, es una estrategia eficaz en tecnologías que proponen la divulgación del conocimiento^(6)^. El apoyo de esos elementos contribuye para que la intervención proyectada sea coherente con su finalidad, incluyendo elementos considerados indispensables para el objetivo que se pretende alcanzar y de esa forma potencializar su efecto^(44)^.

Además del uso de una teoría, la combinación de videos con otros recursos resalta la sinergia entre diferentes abordajes educacionales, como en el caso de sesiones de discusiones^(30)^ y material impreso informativo^(28-29,36)^, que favorece una divulgación de informaciones de forma más atractiva, creando un contacto más próximo con los participantes que tendrán a su disposición un espacio para aclarar sus dudas o un material para lectura y poder profundizar siempre que necesario.

Otro punto a ser considerado es el impacto positivo de las intervenciones audiovisuales en las ISTs. Diversos estudios demostraron que los videos, principalmente los interactivos, además de aumentar el conocimiento, contribuyeron para una reducción de la adquisición de alguna IST^(19)^. Además, también existe la posibilidad de mejorar la disposición de los participantes para los exámenes de clasificación para IST/HIV^(25)^ y adopción de medidas de prevención como la vacunación contra el HPV^(36)^. Una vez que esas tecnologías tienen potencial para captar el interés del público y facilitar el proceso de aprendizaje, ellas mejoran la divulgación de informaciones sobre salud y, por consecuencia, llevan a una adopción de acciones preventivas más asiduas^(22)^.

La respuesta de la eficacia de las intervenciones también es evidenciada en poblaciones específicas, como los usuarios de drogas inyectables^(37)^, hombres que hacen sexo con hombres^(21)^, hombres con deficiencia intelectual^(26)^ y profesionales de la salud^(31)^. Es sabido que personas que se encuentran en alguna situación de vulnerabilidad presentan un riesgo aumentado para IST, por tanto acciones de educación en salud y prevención para esas poblaciones son muy necesarias. Sin embargo, también es reconocida la importancia de garantizar que esas acciones sean ofrecidas de forma continua, ya que el conocimiento puede ser mutable, dependiendo de la situación de vida y salud en que determinados públicos están inseridos^(26)^.

Cuando comparados con sesiones presenciales, los videos se mostraron aceptables y no inferiores en el sentido de llevar a los participantes a la comprensión del conocimiento repasado^(21,27-28)^. Eso demuestra que, una vez aplicados al contexto en el que se presentan, ellos pueden llevar a los mismos beneficios que una sesión expositiva apenas, sin embargo con un potencial mayor para reproducción y consecuente diseminación para un número mayor de personas.

Entre las limitaciones apuntadas en los estudios incluidos, existe la necesidad de considerar: la confiabilidad de los instrumentos de evaluación del conocimiento^(33)^, el tamaño de la muestra, la falta de indicadores clínicos específicos (con el diagnóstico previo) y la necesidad de considerar las barreras culturales y lingüísticas al implementar intervenciones educacionales^(6,23-24)^. En el contexto del HIV, como siendo la IST más trabajada, todavía existe la importancia de abordar el estigma relacionado a la infección dentro de las intervenciones^(31)^.

Los resultados de esta revisión sistemática fortalecen y apoyan el uso de tecnologías audiovisuales para la diseminación de conocimiento sobre las IST, de modo a favorecer el entendimiento, aumentando el nivel de información y contribuyendo para la promoción y prevención de la salud en jóvenes y adultos. Ninguno de los estudios abordó la aplicación de la intervención para adultos mayores o personas que necesitan de alguna accesibilidad (baja visión o audición); este es un vacío que puede ser llenado con nuevos estudios.

La aplicabilidad en la práctica asistencial está en la facilidad de manoseo y uso de esas intervenciones, además del potencial para ser accedidas de forma individual o colectiva, favoreciendo la diseminación de informaciones de forma más amplia y efectiva, así como el cambio de comportamientos y actitudes frente a las ISTs.

Como este estudio no posee límite temporal para la selección de los estudios, es importante reconocer que, a lo largo del tiempo, ocurrieron cambios significativos en el contexto social, cultural y tecnológico que pueden influenciar la interpretación de los resultados. Por ejemplo, la ampliación de la internet y dispositivos digitales transformaron la forma como las personas tienen acceso a las informaciones. Además de eso, hubo avances relacionados al abordaje de las ISTs, lo que pudo haber impactado la percepción de riesgo y la adhesión a medidas preventivas a lo largo del tiempo.

Sin embargo, es importante destacar que los recursos audiovisuales, demostraron ser modos eficaces para aumentar el conocimiento sobre ISTs y HIV/SIDA en diferentes momentos. Un estudio, incluido en esta revisión, realizado en 2005^(34)^, sobre una tesis de doctorado realizada en 2014^(45)^, excluida por no atender a los criterios de elegibilidad, al utilizar la misma intervención con la misma población, produjeron resultados semejantes, con aumentos significativos en el conocimiento. Eso sugiere que, a pesar de los cambios contextuales, las intervenciones bien estructuradas y basadas en recursos audiovisuales pueden mantener su eficacia a lo largo del tiempo.

En relación a las limitaciones, la variabilidad en la calidad metodológica de los estudios incluidos pudieron haber influenciado los resultados, una vez que muchos presentaron alto riesgo de sesgo. Además de eso, la exclusión de estudios no accesibles y de la literatura gris pudieron haber introducido una limitación en el alcance de la investigación, lo pudo haberse reflejado en toda la evidencia disponible sobre el tema. Es posible que la exclusión de esos materiales pueda haber resultado en la omisión de evidencias relevantes, especialmente en contextos en que existe escasez de estudios publicados sobre el tema.

Otra limitación se refiere a los diferentes tipos de intervenciones analizadas, que pueden dificultar la generalización de los resultados. Además de eso, la síntesis narrativa de los resultados, sin un análisis estadístico consolidado, puede limitar la evaluación precisa de los efectos de las intervenciones, destacando la necesidad de realizar nuevos estudios más homogéneos en lo que se refiere al método.

## Conclusión

Las tecnologías educativas audiovisuales, elucidadas por medio de videos, son eficaces en la mejoría del conocimiento, actitudes y prácticas en relación a las ISTs y HIV/SIDA. Al adoptar ese tipo de recurso, se debe considerar el contexto socio educacional en el cual el público a que se destina está inserido, así como aspectos culturales, lingüísticos y de alfabetización en salud. Inclusive sin una comprobación significativa, se sugiere que las intervenciones basadas en modelos teóricos pueden ser bastante más exitosas.

Una vez que la magnitud de los cambios en el conocimiento puede variar, es necesario considerar las características de los participantes, antes de la construcción o selección de la intervención a ser aplicada, para que los elementos esenciales de comunicación con el público estén presentes en la tecnología. Así como hay que considerar la retención del conocimiento y los cambios en los comportamientos de salud en el largo plazo, para garantizar que las informaciones se perpetúen en lo cotidiano de aquellos que recibieron la intervención, al mismo tiempo en que estos también se tornen multiplicadores de ese conocimiento.

## Data Availability

Todos los datos generados o analizados durante este estudio están incluidos en este artículo publicado.
